# What evidence exists on the environmental occurrence and toxic effects of the tire additive 6PPD: a systematic map protocol

**DOI:** 10.1186/s13750-026-00383-y

**Published:** 2026-04-01

**Authors:** Katryna J. Seabrook, Julie E. Adams, Stacey A. Robinson, Markus Brinkmann, Tanya M. Brown, Jonathan K. Challis, Leah Chibwe, Sarah Marteinson, Danielle Philibert, Ryan S. Prosser, Diane M. Orihel

**Affiliations:** 1 School of Environmental Studies, Queen’s University, Kingston, ON K7L 3N6 Canada; 2 Department of Biology, Queen’s University, Kingston, ON Canada; 3 Ecotoxicology and Wildlife Health Division, Wildlife and Landscape Science Directorate, Environment and Climate Change Canada, Ottawa, ON Canada; 4 Toxicology Centre, University of Saskatchewan, Saskatoon, SK Canada; 5 School of Environment and Sustainability, University of Saskatchewan, Saskatoon, SK Canada; 6 Global Institute for Water Security, University of Saskatchewan, Saskatoon, SK Canada; 7 Simon Fraser University, Burnaby, BC Canada; 8 Lethbridge Research and Development Centre, Agriculture and Agri-Food Canada, Lethbridge, Canada; 9 Aquatic Contaminants Research Division, Environment and Climate Change Canada, Burlington, ON Canada; 10 Department of Fisheries and Oceans, Ottawa, ON Canada; 11 Huntsman Marine Science Centre, St. Andrews, NB Canada; 12 School of Environmental Sciences, University of Guelph, Guelph, ON Canada

**Keywords:** Ecotoxicology, Environmental pollution, Tire antioxidant, Tire wear particles, 6PPD-quinone, Water, Sediment, Air, Soil, Biota

## Abstract

**Background:**

The tire additive *N*-(1,3-dimethylbutyl)-*N*ʹ-phenyl-*p*-phenylenediamine (6PPD) is widely produced in large volumes as a rubber antidegradant. This chemical can be released into the environment throughout the lifecycle of rubber products. Recently, 6PPD has become the subject of regulatory interest in some jurisdictions due to its widespread environmental occurrence and the acute toxicity of its transformation product *N*-(1,3-dimethylbutyl)-*N*ʹ-phenyl-*p*-phenylenediamine-quinone (6PPDQ) to some salmonids. As research advances for these emerging contaminants, it is critical to understand whether 6PPD and 6PPDQ concentrations in the environment are high enough to pose a risk to living organisms. Here, we present a protocol to conduct two linked systematic evidence maps related to (1) the occurrence of 6PPD and 6PPDQ in the environment and (2) the effects of 6PPD and 6PPDQ on living organisms. Our objective is to collate information on quantification methods, occurrence data, studied species, and toxicity endpoints. This work will contribute to synthesizing a rapidly expanding body of literature and providing insight into knowledge gaps to direct future work.

**Methods:**

The systematic maps will be developed in accordance with the *Collaboration for Environmental Evidence Guidelines and Standards for Evidence Synthesis in Environmental Management*. A unified search strategy using chemical names, acronyms, identifiers, and trade names for 6PPD and 6PPDQ will be used for both maps. Searches will be conducted in seven databases, and grey literature will be sourced from key websites and Advisory Board input. Search results will be managed in a reference management software and screened at the title/abstract and full-text levels against predefined eligibility criteria based on the Population-Outcome and Population-Exposure-Comparison-Outcome framework for Map 1 and Map 2, respectively. A decision tree designed a priori will guide concurrent screening to determine article eligibility for either or both maps. For all eligible articles, bibliographic and study-specific data will be coded and entered into a searchable database. Both article screening and data coding will be completed by two independent reviewers. Two systematic evidence maps will summarize the evidence base using narrative synthesis, figures, and tables.

**Supplementary Information:**

The online version contains supplementary material available at 10.1186/s13750-026-00383-y.

## Background

The global demand for tires has reached an estimated 3 billion per year [1]. The use and disposal of these tires represent a major source of contaminants entering the environment [2]. During tire wear, chemicals are released into the environment where their presence can have ecological consequences [1]. One such chemical, *N*-(1,3-dimethylbutyl)-*N*ʹ-phenyl-*p*-phenylenediamine (6PPD), is an antidegradant ubiquitously used to prolong tire life by protecting rubber polymers from reactions with both oxygen and ozone [3]. Since the 1960s, 6PPD has been globally produced at high rates. An estimated 130,000 metric tons of 6PPD was produced globally in 2001 [4, 5]. Presently, the production of 6PPD has increased, with an estimated 200,000 metric tons being produced annually in China alone [6]. As a result of its widespread use and subsequent release into the environment, 6PPD has been detected in various environmental compartments, including in samples from road runoff, surface water, sediment, soil, snow, dust and air around the world [7–9]. While the role of 6PPD as an antidegradant in rubber is well characterized, its occurrence and potential effects in the environment as a pollutant remain poorly understood.

The primary source of 6PPD in the environment is from tire wear particles formed during the interaction between vehicle tires and road surfaces [10]. Efforts to recycle used tires for new purposes, such as filler in asphalt and road pavement, a geopolymer to reinforce soils, ground rubber in turfs and playground surfaces [11], may further contribute to the release of 6PPD in the environment. During tire wear, 6PPD migrates toward the tire surface where it reacts with free radicals (i.e., ozone) to form more stable molecules that protect the rubber polymer from degradation [12]. This process can lead to the formation of transformation products on the tire surface, that can then be released into the environment [13]. In 2021, researchers identified an ozone-derived transformation product of 6PPD, *N*-(1,3-dimethylbutyl)-*N*ʹ-phenyl-p-phenylenediamine-quinone (6PPDQ), to be the probable causal agent of mass die-offs of coho salmon in British Columbia (Canada) and Washington (USA) [14]. Consequently, within the last five years, the body of literature on 6PPD and its transformation product, 6PPDQ has grown rapidly [15] and both chemicals have become the subject of regulatory activities [16].

The identification and toxicological properties of 6PPD transformation products were not well studied prior to the identification of 6PPDQ. Due to the unknown mechanisms of action, predicting potential effects of 6PPD and 6PPDQ is challenging, and a species-by-species approach is currently necessary [17]. As such, a growing number of manipulative experiments are investigating the toxicity of 6PPD and 6PPDQ to living organisms across taxonomic groups, including bacteria [18], plants [19, 20], invertebrates [21, 22], and vertebrates [23, 24]. Manipulative experiments have thus far demonstrated a range of effects (sublethal-organismal) across different species, with notable variability in species-specific responses to 6PPD and 6PPDQ exposure [23, 24]. This, in combination with methodological differences between experiments, complicates the integration of results across taxa and the ability to assess the broader ecological consequences of 6PPD and 6PPDQ contamination.

Whereas a number of narrative (e.g., [25]), bibliometric (e.g., [15]) and critical reviews (e.g., [26]) on 6PPD/6PPDQ have been published, no efforts to systematically map the available evidence have been initiated, to the best of our knowledge. Systematic evidence maps are an effective tool to consolidate rapidly expanding evidence bases in an objective, repeatable, and systematic format [27]. To begin answering the critical question of whether 6PPD and 6PPDQ concentrations in the environment are high enough to pose a risk to living organisms, there is a need to synthesize available data regarding the environmental occurrence and toxicity of 6PPD and 6PPDQ. With increasing environmental monitoring and toxicity studies, differences in sampling strategies, analytical methods and reporting practices result in heterogeneous data. By systematically collating and characterizing existing studies, systematic evidence maps support the ability to compare findings across studies, identify key knowledge gaps, prioritize future research directions, and provide a foundation for downstream systematic reviews. Additionally, systematic evidence maps can be repeated (i.e., after voluntary or regulatory changes have been implemented) and used to compare how 6PPD and 6PPDQ occurrence and associated effects may change in response to measures aimed at reducing environmental exposure to 6PPD and 6PPDQ.

## Advisory board engagement 

An Advisory Board was formed to help inform the scope of the systematic maps proposed herein. The Advisory Board reviewed a draft of this protocol, refined and reviewed the map questions, as well as the searching and coding strategies to ensure collected information is relevant to the user group. Members of the Advisory Board were identified as individuals and organizations with expert knowledge on the topic, expert knowledge on systematic methods, or with interest in the formulation and findings of the systematic map and evidence synthesis work [27]. The Advisory Board consists of representatives from academia (University librarians and professors), non-governmental organizations (Raincoast Conservation Foundation and the International Joint Commission), as well as government (Environment Climate Change Canada, Government of Canada). This work is being funded by Environment Climate Change Canada (ECCC) and relevant representatives from ECCC are on the Advisory Board. The Advisory Board will be consulted for ongoing guidance throughout the systematic map and future systematic review processes.

## Objective of the review

**Fig. 1:**
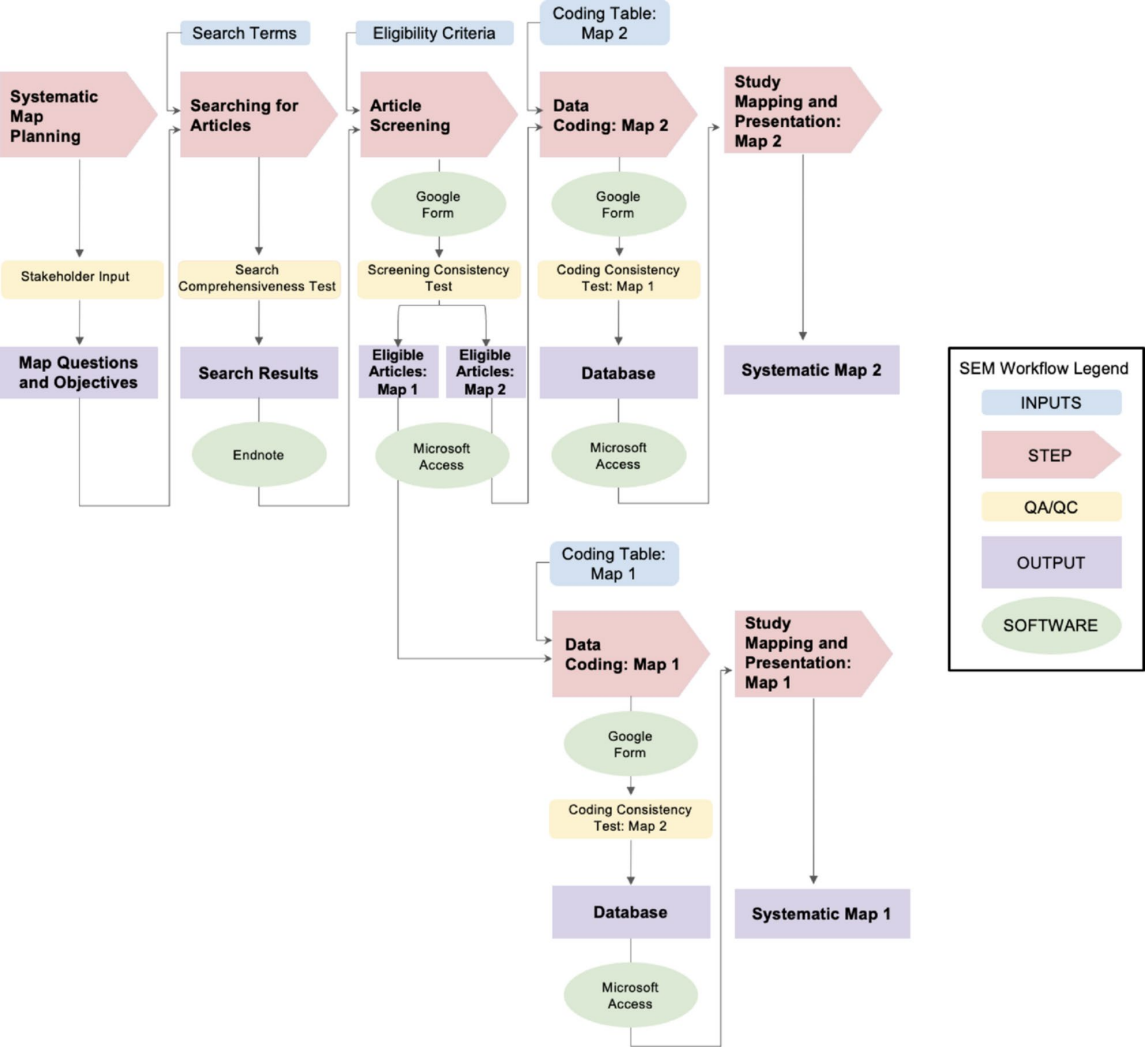
Workflow for systematic evidence mapping (SEM). Key workflow components include inputs (blue rectangles), steps (red arrows), quality assurance/quality control (QA/QC) methods (yellow rectangles), outputs (purple rectangles), and proposed examples of software (green circles). All workflow components are related to Map 1, Map 2, or both. ‘Systematic Map Planning’ and ‘Searching for Articles’ will be conducted for Map 1 and Map 2 simultaneously using the same search terms. The same search terms will be used to capture articles for both maps to reduce duplication of effort, and ensure a transparent and reproducible search process. In this way, ‘Article Screening’ will identify if an article is eligible for Map 1, Map 2, both, or neither. From there, ‘Data Coding’ and ‘Study Mapping and Presentation’ will be conducted for each Map 1 and Map 2 individually

The ultimate question guiding our research program (entitled the ‘6PPD Evidence Synthesis Project’) is “What evidence exists that biota in the environment are exposed to 6PPD and 6PPDQ at concentrations that cause effects in manipulative experiments?” To begin to answer this question, we will produce two systematic maps: one map regarding the occurrence of these compounds in the environment, including biota (hereafter, “Map 1”), and a second map focused on the effects of these compounds on living organisms reported in manipulative experiments (hereafter, “Map 2”). The workflow for our systematic evidence mapping is illustrated in Fig. 1.

Through systematic mapping, we aim to identify research trends and knowledge gaps to specify areas where further primary research is needed and where sufficient evidence exists for systematic review approaches. Therefore, these maps form a foundational step in our 6PPD Evidence Synthesis Project, guiding downstream systematic reviews. Future systematic reviews will bring together the evidence base collated by Map 1 and Map 2 to answer our ultimate question regarding whether levels of 6PPD and 6PPDQ in the environment are above ecotoxicological thresholds established by manipulative experiments.

### Primary questions

The primary question for Map 1 is “What evidence exists globally that biota in the environment are exposed to 6PPD and 6PPDQ?”, of which the Population-Outcome (PO) elements [*sensu* (37)] are defined in Table 1.

**Table 1:** Elements of the primary question for the systematic map regarding the occurrence of 6PPD and 6PPDQ in the environment, including biota (Map 1)

Element	Definition
Population	• Environmental compartments (e.g., water, sediment, soil, air) and biota (e.g., plants, animals, fungi, microorganisms) in all types of ecosystems (e.g., freshwater, marine, estuarine, terrestrial, atmospheric) • Exclude: humans, households, lab-exposed biota, laboratory environments, municipal or industrial waste
Outcome	• Detection or quantification of 6PPD and/or 6PPDQ in environmental compartments and biota (as defined in Population above)

The primary question for Map 2 is “What evidence exists on the effects of 6PPD and 6PPDQ on living organisms in manipulative experiments?”, of which the Population-Exposure-Comparison-Outcome (PECO) elements [*sensu* 37)] are defined in Table 2.

**Table 2:** Elements of the primary question for the systematic map regarding the effects of 6PPD and 6PPDQ on living organisms in manipulative experiments (Map 2)

Element	Definition
Population	• Living organisms (e.g., plants, animals, fungi, microorganisms), as well as proxies for living organisms (e.g., cell lines) in manipulative experiments in the lab or field • Exclude: humans
Exposure	• Experimentally manipulated exposure to 6PPD and/or 6PPDQ by any exposure route (e.g., water, sediment, internal administration) • Exclude: chemical mixtures (e.g., tire leachate, roadway runoff, wastewater, stormwater, biosolids)
Comparator	• Biota not experimentally exposed to 6PPD and/or 6PPDQ in the same experiment
Outcome	• Effects on living organisms at all levels of biological organization (e.g., molecular to community level)

Although for Map 2 we are focusing on 6PPD and 6PPDQ, we acknowledge that the short half-lives of 6PPD and 6PPDQ mean that, in reality, when organisms are experimentally exposed to these compounds, they are inevitably also exposed to their breakdown products. Furthermore, we acknowledge experimental exposures may also involve chemical impurities present in analytical standards (i.e., 6PPDQ present as an impurity in 6PPD standards).

### Secondary questions

The approach we used to develop secondary questions was to ask ‘what, where, who, when, how, and how many?’ concerning the body of evidence available for each systematic map. The formulation of these secondary questions lays the foundation for the data sets we will extract from the literature and collate for the systematic maps. For both maps, these secondary questions are designed to characterize what has been studied, who has conducted the studies, where and when studies were conducted, how analyses or experiments were performed, and how much evidence exists. Together, these questions will allow us to systematically describe global research trends, identify methodological variations, and highlight evidence gaps relevant to monitoring, risk assessment, and future synthesis efforts. The secondary questions for Maps 1 and 2 are provided in Tables 3 and 4, respectively.

**Table 3:** Secondary questions for the systematic map regarding the occurrence of 6PPD and 6PPDQ in the environment, including biota (Map 1)

Approach	Secondary question
What?	What ecosystem (i.e., ecosystem type, sub-ecosystem type) were samples collected from for 6PPD/Q analysis? What environmental compartment (i.e., compartment type, subcompartment type) was sampled for 6PPD/Q analysis? What biota (i.e., biota type, taxon name, life stage, biotic sample type) was sampled for 6PPD/Q analysis? What compounds were analyzed (i.e., 6PPD, 6PPDQ, and any co-contaminants)?
Where?	Where (i.e., media type, media name) was the evidence published? Where (i.e., country, province/state, latitude, longitude, site name) were samples collected for this evidence?
Who?	Who (i.e., author name, author affiliation) published the evidence?
When?	When (i.e., year) was the evidence published? When (i.e., year, month) were samples collected?
How?	How were samples for 6PPD/Q analysis collected (i.e., sampling method) and stored (i.e., storage condition)? How (i.e., method, instrument, standard name) was 6PPD/Q analyzed? How was QA/QC performed (i.e., blanks, replication, detection limit reported)?
How many?	How many publications reported data on 6PPD/Q? How many environmental samples were analyzed for 6PPD/Q? How many biotic samples were analyzed for 6PPD/Q?

Terms in brackets are database fields that we explicitly define in Additional File 3

**Table 4:** Secondary questions for the systematic map regarding the effects of 6PPD and 6PPDQ on living organisms in manipulative experiments (Map 2)

Approach	Secondary question
What?	What living organism (e.g., organism category, organism subcategory, taxon, life stage) was exposed to 6PPD/Q?
Where?	Where (i.e., media type, media name) was the evidence published? Where (i.e., country, province/state, latitude, longitude, site name) were samples collected for this evidence?
Who?	Who (i.e., author name, author affiliation) published the evidence?
When?	When (i.e., year) was the evidence published?
How?	How (i.e., compound name, compound purity, solvent, nominal concentration, exposure route, exposure regime, exposure duration, experimental unit type, experimental unit scale) were living organisms exposed to 6PPD/Q? How (i.e., sample type, method) was exposure to 6PPD/Q characterized? How (i.e., biological organization level, endpoint type, endpoint name) were the effects of 6PPD/Q characterized in living organisms?
How many?	How many publications reported manipulative experiments to assess the effects of 6PPD/Q on biota? How many manipulative experiments on 6PPD/Q were performed? How many living organisms (of each organism category) were assessed for toxicity to 6PPD/Q?

Terms in brackets are database fields that we explicitly define in Additional File 3

## Methods

The systematic maps will follow the Collaboration for Environmental Evidence Guidelines and Standards for Evidence Synthesis in Environmental Management Version 5.1 [28] and will meet the ROSES Reporting standards for Systematic Evidence Syntheses [29] (see Additional File 1).

### Searching for articles

The overall search strategy for collecting articles will include a combination of bibliographic database searches for primary literature and online searches for grey literature. We will also send calls for evidence through social media, email listservs, and society bulletins. While sending calls for evidence through social media is not reproducible, we will report posting details, including location, content, and duration in the Supplementary Information for transparency. A standardized submission form using a Google Form will be used to receive submitted evidence.

### Publication databases to be searched

We will search seven bibliographic databases (Web of Science (All Collections), Scopus, Compendex, SciFinder, GeoRef, GeoBase, and Environment Complete) using Queen’s University library subscriptions. The Web of Science (All Collections) includes the following citation indexes: Web of Science Core Collection, Biological Abstracts, BIOSIS (Citation Index), BIOSIS (Previews), Current Contents Connect, Data Citation Index, Derwent Innovations Index, Grants Index, KCI Korean Journal Database, MEDLINE (PubMed), Policy Citation Index, Preprint Citation Index, ProQuest Dissertations and Theses Citation Index, SciELO Citation Index, and Zoological Records. We will use English language Boolean search modifiers adapted to each database and wildcards where applicable.

### Specialist searches

The collection of grey literature will involve input from Advisory Board contacts as well as searches on Lens.org, open-access databases, and key organizational websites. We aim to collect two distinct types of grey literature: ‘file drawer’ not yet published research and ‘practitioner-generated research’ such as organizational and government reports [30]. We will use two open-access databases, the National Technical Reports Library of US federal technical reports and TRID, international transportation literature from the Transportation Research Board’s Transportation Research Information Services (TRIS) Database and OECD’s Joint Transport Research Centre’s International Transport Research Documentation Database. We will also conduct manual searches of reviews, meta-analysis, book chapters, and modelling articles and manual searches of key organizations’ webpages for ‘Publication’, ‘Resources’, or equivalent pages (see Additional File 2, Table S1).

### Search terms and languages

The proposed search terms include chemical names, acronyms, identifiers, and trade names for 6PPD and 6PPDQ compiled from PubChem and SciFinder (See Additional File 2, Tables S2 and S3). The search will be conducted with all individual search terms combined using the Boolean OR operator. This approach is intentionally broad to generate the most comprehensive search. This approach also enables article searching for both Map 1 and Map 2 to occur simultaneously, reducing duplication of efforts and supporting the clear identification of articles relevant to both Map 1 and Map 2. Search strings and the time of the search will be reported in the systematic maps. In databases where complex search strings are not accepted, modified search strings will be used and reported in the systematic maps. All searches will be conducted in English.

To develop and test the search string, a ‘search comprehensiveness test’ was conducted in Web of Science (All Collections) using a set of 30 articles identified a priori for both Map 1 and Map 2 (see Additional File 2, Table S4). Benchmark articles were strategically selected with input from the review team and the Advisory Board to include both well-known and less widely cited studies that the team considered relevant to the scope of the map primary questions. In this test, all individual search terms listed in Tables S2 and S3 were combined using the Boolean OR operator. This search resulted in a 100% return rate for benchmark articles; however, a large proportion of returned literature was not relevant to answer the map questions (See Additional File 2, Text S1). To refine our search string, we conducted searches with different configurations of the search term categories (See Additional File 2, Table S5) and then used select individual search terms to deduce which were contributing most to the total number of results (See Additional File 2, Table S6 and S7). The titles and abstracts returned from top contributing search terms were screened for relevance to our map questions (See Additional File 2, Table S8 and S9). Based on this exercise, we identified two search terms that could be removed from the search string, improving specificity and reducing the number of irrelevant returns (See Additional File 2, Text S1 for more details on the process to build the search string). The revised search string returned 2,397 results with a 100% return rate for benchmark articles in the ‘search comprehensiveness test’ (See Additional File 2, Table S10).

### Estimating the comprehensiveness of the search

To assess the sensitivity of the search strategy, the ‘search comprehensiveness test’ will be conducted with all databases used in the search, and we will report the proportion of benchmark studies retrieved by our search string and in which databases they were captured.

### Assembling and managing search results

A reference management software (e.g., EndNote) will be used to collate all search results and remove duplicates. Duplicates will be identified manually within the reference management software by sorting records alphabetically by author last name and title. Entries will be considered duplicates when author names, titles, DOIs, and publication dates matched. In cases where both a conference proceeding and the resulting journal article were retrieved, only the journal article will be retained. Similarly, when both a dataset and its associated journal article are retrieved, only the journal article will be retained. This condensed list of search results will then be imported into the screening management software (e.g., NVivo). Extracted data and coding will be archived using a database management system (e.g., Microsoft Access).

## **Article** screening and study eligibility criteria

### Screening process

**Fig. 2:**
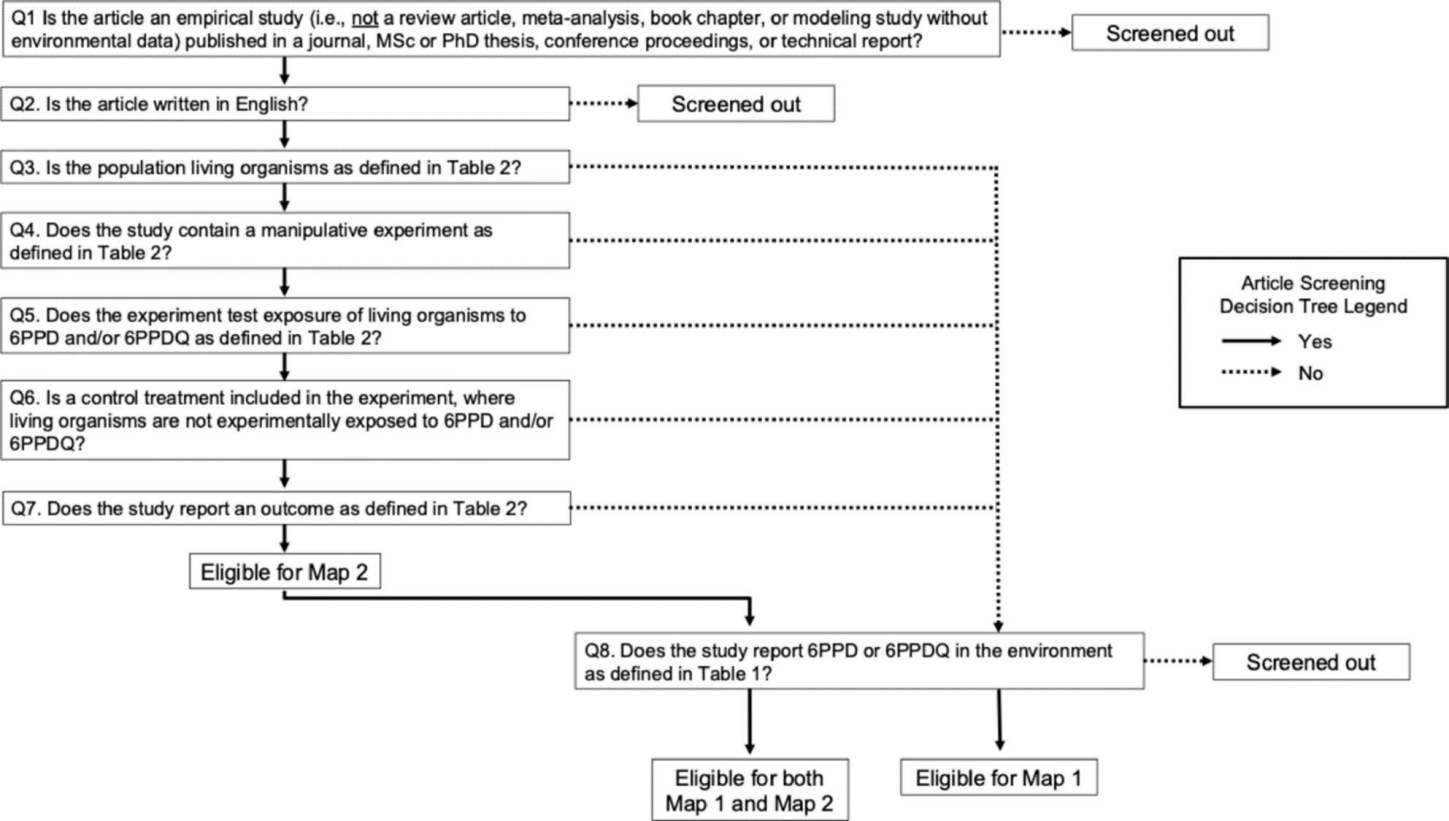
Decision tree to guide screening for article eligibility in the two-stage screening process for both Map 1 and Map 2. Arrows indicate the outcome of each screening question. Solid arrows represent a “Yes” response, permitting articles to proceed to the next screening question, and dashed arrows represent a “No” response. A “No” response to Q3-Q7 will result in exclusion from Map 2 but will permit consideration of eligibility for Map 1 in Q8. This structure allows inclusion in Map 1 only, Map 2 only, or both, depending on the response pathway

A two-stage screening process will be applied, where first the title and abstract (if available), and then the full text, are screened. For both screening stages, the title/abstract and full text will be screened following a decision tree with definitive screening questions (Fig. 2; see Additional File 2, Text S2). Screening of articles will be distributed among the team members. Specifically, at least two randomly selected team members will screen each article independently at both stages (i.e., title and abstract, and full text). However, team members will not screen articles they have authored; instead, an alternate team member will be randomly selected.

To ensure consistency among team members in the application of eligibility criteria for screening, a ‘screening consistency test’ will be used. Specifically, prior to screening articles, at least two team members will independently screen 10 titles/abstracts, where 5 articles are randomly selected from the search list and 5 articles are from the ‘test-list’ (i.e., pre-selected articles that are known to meet eligibility criteria). A similar procedure with a separate list of 10 articles will be checked for consistency of screening at the full-text stage. To test interrater reliability, percent agreement between reviewers and Cohen’s kappa statistic will be calculated and must be ≥ 0.8 (i.e., an acceptable level of agreement between team members according to [31]). Percent agreement will be reported because it is an accessible and easily interpretable measure of consistency. However, because percent agreement does not account for agreement occurring by chance, we will also calculate and report Cohen’s kappa statistic. Given the small sample size (*n* = 10) in the screening consistency tests, confidence intervals for Cohen’s Kappa will be estimated using exact bootstrap methods. Unlike asymptotic intervals, which often yield biased and poorly covered estimates in small samples, the bootstrap approach resamples the observed data to build an empirical distribution of Kappa and derive percentile-based confidence limits. This method avoids normality assumptions and is widely recommended for agreement studies with limited data because it improves coverage accuracy and reduces the risk of misleading conclusions [32]. If the Cohen’s kappa statistic is < 0.8, then the eligibility criteria are re-visited and modified for clarity (as needed) as each disagreement is discussed. This consistency checking process continues until the Cohen’s kappa statistic reaches ≥ 0.8. For each screening consistency test, a subset of different articles will be randomly selected to minimize intra-reviewer recollection of previous screening decisions.

During article screening, all articles will be double screened by two reviewers independently. If during the first stage (i.e., title and abstract screening), there is disagreement between two team members on the screening decision to include, exclude or maybe, the article will proceed to full text screening (along with articles screened in during the first stage) so all available information can be considered for decision making. If a disagreement occurs at the full text screening stage (i.e., exclude or maybe), decisions will be discussed until consensus is reached or if consensus between the two team members cannot be reached, a third randomly selected team member will independently screen the articles in question and then decisions will be discussed until consensus is reached. All screening decisions, including disagreements with consensus discussions, will be tracked and a list of articles excluded at the full text screening stage with reasons for exclusion will be reported in the Supplementary Information.

#### Eligibility criteria

During these screening processes, the relevance and thus inclusion or exclusion eligibility of each article will be determined according to our PO and PECO eligibility criteria (Tables 1 and 2). In addition to the PO and PECO eligibility criteria, articles must also be (i) empirical studies published in a peer-reviewed journal, MSc or PhD thesis, conference proceedings, book chapter, or technical report; and (ii) written in English. Screening decisions at the title and abstract stage will be either: (1) include, (2) exclude, and (3) maybe (e.g., abstract not available, eligibility unclear from title/abstract, or screening reviewers disagree). Titles/abstracts screened as ‘maybe’ will then proceed to full text screening where the screening decisions will be either: (1) include, or (2) exclude. Article screening for Map 1 and Map 2 will be conducted concurrently, such that screening decisions will also specify the appropriate map.

## **Study** validity assessment

A formal validity assessment of included studies will not be conducted during the systematic mapping stage of our research. The purpose of the systematic evidence map is to provide a description of the evidence base, showing the abundance and distribution of evidence across the different elements of our PO and PECO questions, rather than to synthesize and interpret study findings [28]. However, as part of our data coding process, we will code for and extract metadata on aspects of study validity (i.e., study design, timeline, sample size, replication, and randomization details) from included studies that may help describe characteristic qualities of the evidence base. Extracted information at this stage can be used in a full critical appraisal in subsequent systematic review(s) conducted on map outputs.

## **Data** coding strategy

Eligible articles will be assigned a unique “Article ID” that will identify the article throughout the metadata extraction and coding process. Data extraction and coding will be completed using a data collection form (e.g. Google Forms). After collection, the data will be imported into a Microsoft Access database. All eligible articles will be coded for the following information: (i) Article Bibliographic Information; (ii) Study Location Information; and (iii) Chemical Information (see Additional File 3). Articles will be further coded based on variables describing the PO and/or the PECO elements, depending on their eligibility for Map 1 and Map 2, respectively (Tables 1 and 2). Information will be coded in the database using a priori-specified fields (see Additional File 3). N/A will be used as “not applicable”, and NR will be used as “not reported”.

Data coding and metadata extraction from screened-in articles will be distributed among team members, such that at least two randomly selected team members will code each article independently. However, similar to article screening, team members will not assess articles they have authored; instead, an alternate team member will be randomly selected. Any discrepancies will be discussed among team members until a consensus is reached. If consensus between the two team members cannot be reached, a third randomly selected team member will independently code for the data in question, and then decisions are discussed until consensus is reached. All disagreements with consensus discussions will be tracked and reported in the Supplementary Information.

To ensure consistent and repeatable data coding, a random subset of 5% of the screened in articles (or a minimum of 10 articles) will be independently coded by all team members. This subset al.igns with current practice in published systematic maps, which report the use of 10–20 articles for coding consistency checking [33, 34]. This ‘coding consistency test’ will also be used to assess the efficacy and functionality of the data collection form.

## **Study** mapping and presentation

From this protocol, two distinct systematic maps will be generated and published as separate CEE Evidence Syntheses in open-access journals. Each map will include a ROSES flow diagram, narrative synthesis, and quantitative synthesis. Evidence will be synthesized in descriptive statistics, tables, figures, and geographical maps. For example, we anticipate the generation of tables with counts of articles and colour shading to show the magnitude of counts for different coding fields. Anticipated figures will include bar plots with total counts for categorical fields (e.g., study species, environmental compartment, outcome, etc.) and trends over time (counts of articles over time, proportion of articles from different geographical locations, changes in analytical measurement and methodology over time, etc.). Geographical maps will be used to visualize the spatial distribution of 6PPD and 6PPDQ detection efforts from articles included in Map 1 and the location of research groups assessing the toxicity of these contaminants from articles included in Map 2. If applicable, more sophisticated visualization tools such as heat maps will be used. We will produce a searchable database that includes all coded data. Knowledge gaps warranting additional research, under-represented areas, and knowledge clusters where there is sufficient evidence for a full systematic review will be identified.

## Supplementary Information

Below is the link to the electronic supplementary material.

Supplementary Material 1.

Supplementary Material 2.

Supplementary Material 3.

## Data Availability

All data generated or analyzed in this protocol are included in this published article and its supplementary information files.
