# Supplementary material for: What evidence exists on the environmental occurrence and toxic effects of the tire additive 6PPD: a systematic map protocol
Source: Environ Evid. 2026 Apr 1;15:5. doi: 10.1186/s13750-026-00383-y (PMC13200294; doi:10.1186/s13750-026-00383-y)
Supplement: Supplementary file 2 — Supplementary Material 2. [file 13750_2026_383_MOESM2_ESM.docx]

**Supplementary Materials – Additional File 2**

**A systematic map protocol to identify evidence for the environmental occurrence and toxic effects of the tire additive 6PPD**

Katryna J. Seabrook^1*^ (0009-0005-5673-6898), Julie E. Adams^2*^ (0009-0007-3357-4309), Stacey A. Robinson^3^ (0000-0003-4522-0376), Markus Brinkmann^4,5,6^ (0000-0002-4985-263X), Tanya M Brown^7^ (0000-0003-4604-1847), Jonathan K. Challis^8^ (0000-0003-3514-0647), Leah Chibwe^9^ (0000-0003-4870-5192), Sarah Marteinson^10^ (0000-0001-6862-8679), Danielle Philibert^11^ (0000-0001-9763-6432), Ryan S. Prosser^12^ (0000-0001-9087-5748), Diane M. Orihel^1, 2**^ (0000-0002-6933-3650)

^1^ School of Environmental Studies, Queen’s University, Kingston, Ontario, Canada

^2^ Department of Biology, Queen’s University, Kingston, Ontario, Canada

^3^ Ecotoxicology and Wildlife Health Division, Wildlife and Landscape Science Directorate, Environment and Climate Change Canada, Ottawa, Ontario, Canada

^4^ Toxicology Centre, University of Saskatchewan, Saskatoon, Saskatchewan, Canada

^5^ School of Environment and Sustainability, University of Saskatchewan, Saskatoon, Saskatchewan, Canada

^6^ Global Institute for Water Security, University of Saskatchewan, Saskatoon, Saskatchewan, Canada

^7^ Simon Fraser University, Burnaby, British Columbia

^8^ Lethbridge Research and Development Centre, Agriculture and Agri-Food Canada, Lethbridge, Canada

^9^ Aquatic Contaminants Research Division, Environment and Climate Change Canada, Burlington, Ontario, Canada

^10^ Department of Fisheries and Oceans, Ottawa, Ontario Canada

^11^ Huntsman Marine Science Centre, St. Andrews, New Brunswick, Canada

^12^ School of Environmental Sciences, University of Guelph, Guelph, Ontario, Canada

* co-lead authors

** corresponding author

**This file contains:**

Supplementary Tables 1-10

Supplementary Text 1-2

**Table S1.** List of key organization’s webpages that will be used to conduct manual searches.

| **Organization Name** | **Organization Webpage** |
| --- | --- |
| United States Environmental Protection Agency | <https://www.epa.gov/chemical-research/6ppd-quinone> |
| Washington State Department of Ecology | <https://ecology.wa.gov/waste-toxics/reducing-toxic-chemicals/addressing-priority-toxic-chemicals/6ppd> |
| Tire Stewardship British Columbia | <https://tsbc.ca/6ppd-in-tires-what-we-know-so-far/> |
| Tire and Rubber Association of Canada | <https://tracanada.ca/industry-news/tire-manufacturers-consortium-6ppd-alternatives-analysis-report-receives-notice-of-compliance-from-california-dtsc-clearing-way-for-stage-2/> |
| The Tire Industry Project | <https://tireindustryproject.org/faq/what-is-6ppd-quinone/> |
| Interstate Technology & Regulatory Council | [https://6ppd.itrcweb.org/](https://6ppd.itrcweb.org/)) |
| Department of Toxic Substances Control, State of California | https://dtsc.ca.gov/ |
| European Chemicals Agency | https://echa.europa.eu/home |

**Table S2.** Proposed search terms for 6PPD.

| **Name (Search Term: TOPIC)** | **Category** | **Notes** |
| --- | --- | --- |
| [6PPD](https://www.ncbi.nlm.nih.gov/pcsubstance/?term=%226PPD%22%5BCompleteSynonym%5D%20AND%2013101%5BStandardizedCID%5D) | acronym |  |
| [DBDA](https://www.ncbi.nlm.nih.gov/pcsubstance/?term=%22DBDA%22%5BCompleteSynonym%5D%20AND%2013101%5BStandardizedCID%5D) | acronym | in PubChem & SciFinder |
| [DMBPD](https://www.ncbi.nlm.nih.gov/pcsubstance/?term=%22DMBPD%22%5BCompleteSynonym%5D%20AND%2013101%5BStandardizedCID%5D) | acronym | in PubChem & SciFinder |
| DPPDA | acronym |  |
| [(4-anilino-phenyl)-(1,3-dimethyl-butyl)-amine](https://www.ncbi.nlm.nih.gov/pcsubstance/?term=%22(4-anilino-phenyl)-(1%2C3-dimethyl-butyl)-amine%22%5BCompleteSynonym%5D%20AND%2013101%5BStandardizedCID%5D) | chemical name |  |
| [1,4-Benzenediamine](https://www.ncbi.nlm.nih.gov/pcsubstance/?term=%221%2C4-Benzenediamine%2C%20N1-(1%2C3-dimethylbutyl)-N4-phenyl-%22%5BCompleteSynonym%5D%20AND%2013101%5BStandardizedCID%5D) | chemical name |  |
| [1,4-Benzenediamine, N-(1,3-dimethylbutyl)-N'-phenyl-](https://www.ncbi.nlm.nih.gov/pcsubstance/?term=%221%2C4-Benzenediamine%2C%20N-(1%2C3-dimethylbutyl)-N%27-phenyl-%22%5BCompleteSynonym%5D%20AND%2013101%5BStandardizedCID%5D) | chemical name |  |
| 1,4-Benzenediamine, N-(1,3-dimethylbutyl)-N′-phenyl- (9CI) | chemical name | in SciFinder |
| 1-N-(4-methylpentan-2-yl)-1-N-phenylbenzene-1,4-diamine | chemical name |  |
| 1-N-(4-methylpentan-2-yl)-4-N-phenylbenzene-1,4-diamine | chemical name | in PubChem & SciFinder |
| [4-(1,3-Dimethylbutyl)amino-diphenylamine](https://www.ncbi.nlm.nih.gov/pcsubstance/?term=%224-(1%2C3-Dimethylbutyl)amino-diphenylamine%22%5BCompleteSynonym%5D%20AND%2013101%5BStandardizedCID%5D) | chemical name |  |
| [4-(1,3-dimethylbutylamino)diphenylamine](https://www.ncbi.nlm.nih.gov/pcsubstance/?term=%224-(1%2C3-dimethylbutylamino)diphenylamine%22%5BCompleteSynonym%5D%20AND%2013101%5BStandardizedCID%5D) | chemical name | in PubChem & SciFinder |
| 4-(Dimethylbutylamino)diphenylamine | chemical name |  |
| [4-[(4-Methyl-2-pentyl)amino]diphenylamine](https://www.ncbi.nlm.nih.gov/pcsubstance/?term=%224-%5B(4-Methyl-2-pentyl)amino%5Ddiphenylamine%22%5BCompleteSynonym%5D%20AND%2013101%5BStandardizedCID%5D) | chemical name |  |
| [4-N-(4-methylpentan-2-yl)-1-N-phenylbenzene-1,4-diamine](https://www.ncbi.nlm.nih.gov/pcsubstance/?term=%224-N-(4-methylpentan-2-yl)-1-N-phenylbenzene-1%2C4-diamine%22%5BCompleteSynonym%5D%20AND%2013101%5BStandardizedCID%5D) | chemical name |  |
| [n-(1,3-dimethyl butyl)-n'-phenyl-p-phenylenediamine](https://www.ncbi.nlm.nih.gov/pcsubstance/?term=%22n-(1%2C3-dimethyl%20butyl)-n%27-phenyl-p-phenylenediamine%22%5BCompleteSynonym%5D%20AND%2013101%5BStandardizedCID%5D) | chemical name |  |
| [N-(1,3-Dimethylbutyl)-N/'-phenyl-p-phenylenediamine](https://www.ncbi.nlm.nih.gov/pcsubstance/?term=%22N-(1%2C3-Dimethylbutyl)-N%2F%27-phenyl-p-phenylenediamine%22%5BCompleteSynonym%5D%20AND%2013101%5BStandardizedCID%5D) | chemical name | , N/' |
| [N-(1,3-Dimethylbutyl)-N\'-phenyl-p-phenylenediamine](https://www.ncbi.nlm.nih.gov/pcsubstance/?term=%22N-(1%2C3-Dimethylbutyl)-N%5C%27-phenyl-p-phenylenediamine%22%5BCompleteSynonym%5D%20AND%2013101%5BStandardizedCID%5D) | chemical name |  |
| [n-(1,3-dimethylbutyl)-n'-phenyl-1,4-benzenediamine](https://www.ncbi.nlm.nih.gov/pcsubstance/?term=%22n-(1%2C3-dimethylbutyl)-n%27-phenyl-1%2C4-benzenediamine%22%5BCompleteSynonym%5D%20AND%2013101%5BStandardizedCID%5D) | chemical name | in PubChem & SciFinder |
| [N-(1,3-Dimethylbutyl)-N'-phenyl-1,4-phenylenediamine](https://www.ncbi.nlm.nih.gov/pcsubstance/?term=%22N-(1%2C3-Dimethylbutyl)-N%27-phenyl-1%2C4-phenylenediamine%22%5BCompleteSynonym%5D%20AND%2013101%5BStandardizedCID%5D) | chemical name | in PubChem & SciFinder |
| [N-(1,3-dimethylbutyl)-N'-phenylbenzene-1,4-diamine](https://www.ncbi.nlm.nih.gov/pcsubstance/?term=%22N-(1%2C3-dimethylbutyl)-N%27-phenylbenzene-1%2C4-diamine%22%5BCompleteSynonym%5D%20AND%2013101%5BStandardizedCID%5D) | chemical name |  |
| [N-(1,3-Dimethylbutyl)-N'-phenyl-p-phenylenediamine](https://www.ncbi.nlm.nih.gov/pcsubstance/?term=%22N-(1%2C3-Dimethylbutyl)-N%27-phenyl-p-phenylenediamine%22%5BCompleteSynonym%5D%20AND%2013101%5BStandardizedCID%5D) | chemical name | in PubChem & SciFinder |
| N-(4-Methyl-2-pentyl)-N-phenyl-1,4-benzenediamine | chemical name |  |
| N-(4-Methyl-2-pentyl)-N-phenyl-1,4-diaminobenzene | chemical name |  |
| [N-(4-Methyl-2-pentyl)-N'-phenyl-1,4-phenylenediamine](https://www.ncbi.nlm.nih.gov/pcsubstance/?term=%22N-(4-Methyl-2-pentyl)-N%27-phenyl-1%2C4-phenylenediamine%22%5BCompleteSynonym%5D%20AND%2013101%5BStandardizedCID%5D) | chemical name |  |
| [N-(4-Methyl-2-pentyl)-N'-phenyl-p-phenylenediamine](https://www.ncbi.nlm.nih.gov/pcsubstance/?term=%22N-(4-Methyl-2-pentyl)-N%27-phenyl-p-phenylenediamine%22%5BCompleteSynonym%5D%20AND%2013101%5BStandardizedCID%5D) | chemical name | in PubChem & SciFinder |
| N1-(1,3-Dimethylbutyl)-N4-phenyl-1,4-benzenediamine | chemical name |  |
| *N*1-(1,3-Dimethylbutyl)-*N*4-phenyl-1,4-benzenediamine (ACI) | chemical name | in SciFinder |
| N1-(4-methylpentan-2-yl)-N4-phenylbenzene-1,4-diamine | chemical name |  |
| N-1,3-dimethylbutyl-N`-phenyl-p-phenylenediamine | chemical name |  |
| [N-1,3-Dimethylbutyl-N'-phenyl-p-phenylendiamine](https://www.ncbi.nlm.nih.gov/pcsubstance/?term=%22N-1%2C3-Dimethylbutyl-N%27-phenyl-p-phenylendiamine%22%5BCompleteSynonym%5D%20AND%2013101%5BStandardizedCID%5D) | chemical name | * phenylenEdiamine mispelled |
| [N-1,3-Dimethylbutyl-N'-phenyl-p-phenylenediamine](https://www.ncbi.nlm.nih.gov/pcsubstance/?term=%22N-1%2C3-Dimethylbutyl-N%27-phenyl-p-phenylenediamine%22%5BCompleteSynonym%5D%20AND%2013101%5BStandardizedCID%5D) | chemical name |  |
| N-1,3-dimethylbutyl-N'-phenyl-p-phenylene-diamine | chemical name |  |
| N-Dimethylbutyl-N'-phenyl-p-phenylendiamine | chemical name |  |
| [N-Phenyl-N'-(1,3-dimethyl butyl)-para-phenylenediamine](https://www.ncbi.nlm.nih.gov/pcsubstance/?term=%22N-Phenyl-N%27-(1%2C3-dimethyl%20butyl)-para-phenylenediamine%22%5BCompleteSynonym%5D%20AND%2013101%5BStandardizedCID%5D) | chemical name |  |
| [N-PHENYL-N'-(1,3-DIMETHYL BUTYL)-PARA-PHENYLENEDIAMINE](https://www.ncbi.nlm.nih.gov/pcsubstance/?term=%22N-PHENYL-N%27-(1%2C3-DIMETHYL%20BUTYL)-PARA-PHENYLENEDIAMINE%20%5BHSDB%5D%22%5BCompleteSynonym%5D%20AND%2013101%5BStandardizedCID%5D) | chemical name |  |
| [n-phenyl-n'-(1,3-dimethylbutyl)-1,4-phenylenediamine](https://www.ncbi.nlm.nih.gov/pcsubstance/?term=%22n-phenyl-n%27-(1%2C3-dimethylbutyl)-1%2C4-phenylenediamine%22%5BCompleteSynonym%5D%20AND%2013101%5BStandardizedCID%5D) | chemical name | in PubChem & SciFinder |
| [N-PHENYL-N'-(1,3-DIMETHYLBUTYL)-P-PHENYL DIAMINE](https://www.ncbi.nlm.nih.gov/pcsubstance/?term=%22N-PHENYL-N%27-(1%2C3-DIMETHYLBUTYL)-P-PHENYL%20DIAMINE%22%5BCompleteSynonym%5D%20AND%2013101%5BStandardizedCID%5D) | chemical name |  |
| [N-Phenyl-N'-(1,3-dimethylbutyl)-p-phenylenediamine](https://www.ncbi.nlm.nih.gov/pcsubstance/?term=%22N-Phenyl-N%27-(1%2C3-dimethylbutyl)-p-phenylenediamine%22%5BCompleteSynonym%5D%20AND%2013101%5BStandardizedCID%5D) | chemical name | in PubChem & SciFinder |
| [p-Phenylenediamine, N-(1,3-dimethylbutyl)-N'-phenyl-](https://www.ncbi.nlm.nih.gov/pcsubstance/?term=%22p-Phenylenediamine%2C%20N-(1%2C3-dimethylbutyl)-N%27-phenyl-%22%5BCompleteSynonym%5D%20AND%2013101%5BStandardizedCID%5D) | chemical name |  |
| *p*-Phenylenediamine, *N*-(1,3-dimethylbutyl)-*N*′-phenyl- (7CI, 8CI) | chemical name | in SciFinder |
| 2921519090 | identifier | Hs Code |
| "ISCS number 1635" | identifier | ISCS Number |
| "UN number 3077" | identifier | UN Number |
| [50809-58-0](https://www.ncbi.nlm.nih.gov/pcsubstance/?term=%2250809-58-0%22%5BCompleteSynonym%5D%20AND%2013101%5BStandardizedCID%5D) | identifier | deprecated CAS |
| [76600-84-5](https://www.ncbi.nlm.nih.gov/pcsubstance/?term=%2276600-84-5%22%5BCompleteSynonym%5D%20AND%2013101%5BStandardizedCID%5D) | identifier | deprecated CAS |
| [793-24-8](https://www.ncbi.nlm.nih.gov/pcsubstance/?term=%22793-24-8%22%5BCompleteSynonym%5D%20AND%2013101%5BStandardizedCID%5D) | identifier | CAS |
| [AKOS015901311](https://www.ncbi.nlm.nih.gov/pcsubstance/?term=%22AKOS015901311%22%5BCompleteSynonym%5D%20AND%2013101%5BStandardizedCID%5D) | identifier | unknown (Turkish company?) |
| [BRN 2215491](https://www.ncbi.nlm.nih.gov/pcsubstance/?term=%22BRN%202215491%22%5BCompleteSynonym%5D%20AND%2013101%5BStandardizedCID%5D) | identifier |  |
| [CAS-793-24-8](https://www.ncbi.nlm.nih.gov/pcsubstance/?term=%22CAS-793-24-8%22%5BCompleteSynonym%5D%20AND%2013101%5BStandardizedCID%5D) | identifier | CAS |
| [CCRIS 2352](https://www.ncbi.nlm.nih.gov/pcsubstance/?term=%22CCRIS%202352%22%5BCompleteSynonym%5D%20AND%2013101%5BStandardizedCID%5D) | identifier | unknown |
| [CCRIS 4801](https://www.ncbi.nlm.nih.gov/pcsubstance/?term=%22CCRIS%204801%22%5BCompleteSynonym%5D%20AND%2013101%5BStandardizedCID%5D) | identifier | unknown |
| [CHEMBL1558796](https://www.ncbi.nlm.nih.gov/pcsubstance/?term=%22CHEMBL1558796%22%5BCompleteSynonym%5D%20AND%2013101%5BStandardizedCID%5D) | identifier | ChEMBL ID |
| CI | identifier | in SciFinder |
| [CS-W012405](https://www.ncbi.nlm.nih.gov/pcsubstance/?term=%22CS-W012405%22%5BCompleteSynonym%5D%20AND%2013101%5BStandardizedCID%5D) | identifier | catalog number from SmallMolecules.com., ChemScene |
| [D3331](https://www.ncbi.nlm.nih.gov/pcsubstance/?term=%22D3331%22%5BCompleteSynonym%5D%20AND%2013101%5BStandardizedCID%5D) | identifier | external ID |
| [DTXCID605114](https://www.ncbi.nlm.nih.gov/pcsubstance/?term=%22DTXCID605114%22%5BCompleteSynonym%5D%20AND%2013101%5BStandardizedCID%5D) | identifier | dsstox compound id, US EPA |
| [DTXSID9025114](https://www.ncbi.nlm.nih.gov/pcsubstance/?term=%22DTXSID9025114%22%5BCompleteSynonym%5D%20AND%2013101%5BStandardizedCID%5D) | Identifier | - EPA CompTox |
| [E76147](https://www.ncbi.nlm.nih.gov/pcsubstance/?term=%22E76147%22%5BCompleteSynonym%5D%20AND%2013101%5BStandardizedCID%5D) | identifier | AstaTech Inc |
| [EC 212-344-0](https://www.ncbi.nlm.nih.gov/pcsubstance/?term=%22EC%20212-344-0%22%5BCompleteSynonym%5D%20AND%2013101%5BStandardizedCID%5D) | identifier | - ECHA, European Chemicals Agency |
| [EINECS 212-344-0](https://www.ncbi.nlm.nih.gov/pcsubstance/?term=%22EINECS%20212-344-0%22%5BCompleteSynonym%5D%20AND%2013101%5BStandardizedCID%5D) | identifier | - ECHA, European Chemicals Agency |
| [GG-0240](https://www.ncbi.nlm.nih.gov/pcsubstance/?term=%22GG-0240%22%5BCompleteSynonym%5D%20AND%2013101%5BStandardizedCID%5D) | identifier | external ID (in google does not return any relevant searches) |
| [HJD0U67PS1](https://www.ncbi.nlm.nih.gov/pcsubstance/?term=%22HJD0U67PS1%22%5BCompleteSynonym%5D%20AND%2013101%5BStandardizedCID%5D) | Identifier | - FDA UNII |
| [HSDB 5755](https://www.ncbi.nlm.nih.gov/pcsubstance/?term=%22HSDB%205755%22%5BCompleteSynonym%5D%20AND%2013101%5BStandardizedCID%5D) | identifier | unknown |
| [MFCD00072248](https://www.ncbi.nlm.nih.gov/pcsubstance/?term=%22MFCD00072248%22%5BCompleteSynonym%5D%20AND%2013101%5BStandardizedCID%5D) | identifier | - MDL number |
| [NCGC00091548-01](https://www.ncbi.nlm.nih.gov/pcsubstance/?term=%22NCGC00091548-01%22%5BCompleteSynonym%5D%20AND%2013101%5BStandardizedCID%5D) | identifier | external ID, NCATS |
| [NCGC00091548-02](https://www.ncbi.nlm.nih.gov/pcsubstance/?term=%22NCGC00091548-02%22%5BCompleteSynonym%5D%20AND%2013101%5BStandardizedCID%5D) | identifier | external ID, NCATS |
| [NCGC00091548-03](https://www.ncbi.nlm.nih.gov/pcsubstance/?term=%22NCGC00091548-03%22%5BCompleteSynonym%5D%20AND%2013101%5BStandardizedCID%5D) | identifier | external ID, NCATS |
| [NCGC00258444-01](https://www.ncbi.nlm.nih.gov/pcsubstance/?term=%22NCGC00258444-01%22%5BCompleteSynonym%5D%20AND%2013101%5BStandardizedCID%5D) | identifier | unknown |
| [NS00003932](https://www.ncbi.nlm.nih.gov/pcsubstance/?term=%22NS00003932%22%5BCompleteSynonym%5D%20AND%2013101%5BStandardizedCID%5D) | identifier | SusDat ID - EU/UNDP project, 2021 |
| [Q-201440](https://www.ncbi.nlm.nih.gov/pcsubstance/?term=%22Q-201440%22%5BCompleteSynonym%5D%20AND%2013101%5BStandardizedCID%5D) | identifier | supplier catalog, Biosynth |
| [Q27279957](https://www.ncbi.nlm.nih.gov/pcsubstance/?term=%22Q27279957%22%5BCompleteSynonym%5D%20AND%2013101%5BStandardizedCID%5D) | identifier | wikidata |
| [SCHEMBL39447](https://www.ncbi.nlm.nih.gov/pcsubstance/?term=%22SCHEMBL39447%22%5BCompleteSynonym%5D%20AND%2013101%5BStandardizedCID%5D) | identifier | unknown |
| [Tox21_200890](https://www.ncbi.nlm.nih.gov/pcsubstance/?term=%22Tox21_200890%22%5BCompleteSynonym%5D%20AND%2013101%5BStandardizedCID%5D) | identifier | unknown |
| [UNII-HJD0U67PS1](https://www.ncbi.nlm.nih.gov/pcsubstance/?term=%22UNII-HJD0U67PS1%22%5BCompleteSynonym%5D%20AND%2013101%5BStandardizedCID%5D) | identifier | - FDA UNII |
| [UOP 562](https://www.ncbi.nlm.nih.gov/pcsubstance/?term=%22UOP%20562%22%5BCompleteSynonym%5D%20AND%2013101%5BStandardizedCID%5D) | identifier | unknown |
| [UOP 588](https://www.ncbi.nlm.nih.gov/pcsubstance/?term=%22UOP%20588%22%5BCompleteSynonym%5D%20AND%2013101%5BStandardizedCID%5D) | identifier | unknown |
| "Accinox ZC" | trade name | in SciFinder |
| ["Akrochem antiozonant pd-2"](https://www.ncbi.nlm.nih.gov/pcsubstance/?term=%22Akrochem%20antiozonant%20pd-2%22%5BCompleteSynonym%5D%20AND%2013101%5BStandardizedCID%5D) | trade name | Akrochem corporation |
| ["Antage 6C"](https://www.ncbi.nlm.nih.gov/pcsubstance/?term=%22Antage%206C%22%5BCompleteSynonym%5D%20AND%2013101%5BStandardizedCID%5D) | trade name | in PubChem & SciFinder |
| "Antigene 6C" | trade name | in SciFinder |
| ["Antioxidant 4020"](https://www.ncbi.nlm.nih.gov/pcsubstance/?term=%22Antioxidant%204020%22%5BCompleteSynonym%5D%20AND%2013101%5BStandardizedCID%5D) | trade name | in PubChem & SciFinder |
| "Antioxidant 6C" | trade name | in SciFinder |
| ["Antioxidant CD 13"](https://www.ncbi.nlm.nih.gov/pcsubstance/?term=%22Antioxidant%20CD%2013%22%5BCompleteSynonym%5D%20AND%2013101%5BStandardizedCID%5D) | trade name | in PubChem & SciFinder |
| ["Antioxidant cd"](https://www.ncbi.nlm.nih.gov/pcsubstance/?term=%22Antioxidant%20cd%22%5BCompleteSynonym%5D%20AND%2013101%5BStandardizedCID%5D) | trade name | unknown - cd may stand for cyclodextrin or cadmium |
| "Antioxidant PD 2" | trade name | in SciFinder |
| ["Antozite 67"](https://www.ncbi.nlm.nih.gov/pcsubstance/?term=%22Antozite%2067%22%5BCompleteSynonym%5D%20AND%2013101%5BStandardizedCID%5D) | trade name | in PubChem & SciFinder |
| ["Antozite 67F"](https://www.ncbi.nlm.nih.gov/pcsubstance/?term=%22Antozite%2067F%22%5BCompleteSynonym%5D%20AND%2013101%5BStandardizedCID%5D) | trade name | in PubChem & SciFinder |
| ["Diafen 13"](https://www.ncbi.nlm.nih.gov/pcsubstance/?term=%22Diafen%2013%22%5BCompleteSynonym%5D%20AND%2013101%5BStandardizedCID%5D) | trade name | in PubChem & SciFinder |
| ["Diafen FDMB"](https://www.ncbi.nlm.nih.gov/pcsubstance/?term=%22Diafen%20FDMB%22%5BCompleteSynonym%5D%20AND%2013101%5BStandardizedCID%5D) | trade name | in PubChem & SciFinder |
| ["Dusantox 6PPD"](https://www.ncbi.nlm.nih.gov/pcsubstance/?term=%22Dusantox%206PPD%22%5BCompleteSynonym%5D%20AND%2013101%5BStandardizedCID%5D) | trade name | in PubChem & SciFinder |
| ["Flexzone 7F"](https://www.ncbi.nlm.nih.gov/pcsubstance/?term=%22Flexzone%207F%22%5BCompleteSynonym%5D%20AND%2013101%5BStandardizedCID%5D) | trade name | in PubChem & SciFinder |
| ["Flexzone 7L"](https://www.ncbi.nlm.nih.gov/pcsubstance/?term=%22Flexzone%207L%22%5BCompleteSynonym%5D%20AND%2013101%5BStandardizedCID%5D) | trade name | in PubChem & SciFinder |
| "Flexzone 7P" | trade name | in SciFinder |
| ["Forte 6C"](https://www.ncbi.nlm.nih.gov/pcsubstance/?term=%22Forte%206C%22%5BCompleteSynonym%5D%20AND%2013101%5BStandardizedCID%5D) | trade name | in PubChem & SciFinder |
| "Kumanox 13" | trade name | in SciFinder |
| "Kumanox 13C" | trade name | in SciFinder |
| "Luvomaxx 6PPD" | trade name | in SciFinder |
| "Nocceler 6C" | trade name | in SciFinder |
| ["Nocrac 6C"](https://www.ncbi.nlm.nih.gov/pcsubstance/?term=%22Nocrac%206C%22%5BCompleteSynonym%5D%20AND%2013101%5BStandardizedCID%5D) | trade name | in PubChem & SciFinder |
| ["Nocrane 6C"](https://www.ncbi.nlm.nih.gov/pcsubstance/?term=%22Nocrane%206C%22%5BCompleteSynonym%5D%20AND%2013101%5BStandardizedCID%5D) | trade name |  |
| ["Nocrane 7 L"](https://www.ncbi.nlm.nih.gov/pcsubstance/?term=%22Nocrane%207%20L%22%5BCompleteSynonym%5D%20AND%2013101%5BStandardizedCID%5D) | trade name |  |
| ["Ozonon 6C"](https://www.ncbi.nlm.nih.gov/pcsubstance/?term=%22Ozonon%206C%22%5BCompleteSynonym%5D%20AND%2013101%5BStandardizedCID%5D) | trade name |  |
| ["Ozonone 6C"](https://www.ncbi.nlm.nih.gov/pcsubstance/?term=%22Ozonone%206C%22%5BCompleteSynonym%5D%20AND%2013101%5BStandardizedCID%5D) | trade name | in PubChem & SciFinder |
| "PD 2" | trade name | in SciFinder |
| ["Permanax 120"](https://www.ncbi.nlm.nih.gov/pcsubstance/?term=%22Permanax%20120%22%5BCompleteSynonym%5D%20AND%2013101%5BStandardizedCID%5D) | trade name | in PubChem & SciFinder |
| ["Permanax 6PPD"](https://www.ncbi.nlm.nih.gov/pcsubstance/?term=%22Permanax%206PPD%22%5BCompleteSynonym%5D%20AND%2013101%5BStandardizedCID%5D) | trade name | in PubChem & SciFinder |
| "Pilflex 13" | trade name | in SciFinder |
| "Rubatan BF" | trade name |  |
| ["Santoflex 13"](https://www.ncbi.nlm.nih.gov/pcsubstance/?term=%22Santoflex%2013%22%5BCompleteSynonym%5D%20AND%2013101%5BStandardizedCID%5D) | trade name | Santoflex 134PD is a mix of 6PPD and 7PPD, in PubChem & SciFinder |
| ["Santoflex 13F"](https://www.ncbi.nlm.nih.gov/pcsubstance/?term=%22Santoflex%2013F%22%5BCompleteSynonym%5D%20AND%2013101%5BStandardizedCID%5D) | trade name | in PubChem & SciFinder |
| ["Santoflex 6PPD"](https://www.ncbi.nlm.nih.gov/pcsubstance/?term=%22Santoflex%206PPD%22%5BCompleteSynonym%5D%20AND%2013101%5BStandardizedCID%5D) | trade name | in PubChem & SciFinder |
| "Stangard 6PPD" | trade name | in SciFinder |
| "Sunsine 6PPD" | trade name | in SciFinder |
| "UOP 562" | trade name | in SciFinder |
| "UOP 588" | trade name | in SciFinder |
| "Vulkanox 4020" | trade name | in SciFinder |
| "Vulkanox 4020LG" | trade name | in SciFinder |
| ["Wingstay 300"](https://www.ncbi.nlm.nih.gov/pcsubstance/?term=%22Wingstay%20300%22%5BCompleteSynonym%5D%20AND%2013101%5BStandardizedCID%5D) | trade name | in PubChem & SciFinder |
| PX-13 | trade name | <https://worldtradescanner.com/Rubber%20Chemical%20Final%20Findings-6PPD.pdf> |
| ["CD 13"](https://www.ncbi.nlm.nih.gov/pcsubstance/?term=%22CD%2013%22%5BCompleteSynonym%5D%20AND%2013101%5BStandardizedCID%5D) | unknown | use as Antioxidant CD13 as CD13 itself returns unrelated, in PubChem & SciFinder |
| [NCI-C56315](https://www.ncbi.nlm.nih.gov/pcsubstance/?term=%22NCI-C56315%22%5BCompleteSynonym%5D%20AND%2013101%5BStandardizedCID%5D) | unknown |  |

**Table S3.** Proposed search terms for 6PPDQ

| **Name** | **Category** | **Notes** |
| --- | --- | --- |
| 6PPD* | acronym |  |
| 6PPDQ | acronym |  |
| 6PPD-Q | acronym |  |
| 6PPD-q | acronym |  |
| 6PPD-quinone | acronym |  |
| [154926030](https://pubchem.ncbi.nlm.nih.gov/compound/154926030) | identifier | PUBCHEM # |
| 2-((4-Methylpentan-2-yl)amino)-5-(phenylamino)cyclohexa-2,5-diene-1,4-dione | chemical name | in PubChem & SciFinder |
| 2,5-Cyclohexadiene-1,4-dione, 2-[(1,3-dimethylbutyl)amino]-5-(phenylamino)- | chemical name |  |
| 2-[(1,3-Dimethylbutyl)amino]-5-(phenylamino)-2,5-cyclohexadiene-1,4-dione | chemical name | ACI |
| 2754428-18-5 | identifier | CAS |
| [DTXSID301034849](https://comptox.epa.gov/dashboard/chemical/details/DTXSID301034849) | identifier | EPA CompTox |
| G8MFB8G7B6 | identifier | FDA UNII |
| N-(1,3-Dimethylbutyl)-N′-phenyl-p-phenylenediamine quinone | chemical name |  |

**Table S4.** List of benchmark articles for Map 1 and Map 2 ‘search comprehensiveness test’.

| **Article - Bibliometric Information** | **Eligibility** | **Captured in WoS (All Collections) search**  August 5, 2025 |
| --- | --- | --- |
| (1) Abudumutailifu M, Li CZ, Xiong HP, Liu SH, Li CL, Cai DM, et al. Tire Rubber-Derived Cyclic Amines in Urban Ambient Particulate Matter in Shanghai. ACS Earth Space Chem. 2025:11. | Map 1 | Yes |
| (2) Cao GD, Wang W, Zhang J, Wu PF, Zhao XC, Yang Z, et al. New evidence of rubber-derived quinones in water, air, and soil. Environ Sci Technol. 2022;56(7):4142-50. | Map 1 | Yes |
| (3) Challis JK, Popick H, Prajapati S, Harder P, Giesy JP, McPhedran K, Brinkmann M. Occurrences of tire rubber-derived contaminants in cold-climate urban runoff. Environmental Science & Technology Letters. 2021;8(11):961-7. | Map 1 | Yes |
| (4) Di SS, Xu HG, Yu YD, Qi PP, Wang ZW, Liu ZZ, et al. Environmentally relevant concentrations of S-6PPD-Quinone caused more serious hepatotoxicity than R-enantiomer and racemate in Oncorhynchus mykiss. Environ Sci Technol. 2024;58(40):17617-28. | Map 2 | Yes |
| (5) Fang CL, Fang LY, Di SS, Yu YD, Wang XQ, Wang CH, Jin YX. Characterization of N-(1,3-dimethylbutyl)-N'-phenyl-p-phenylenediamine (6PPD)-induced cardiotoxicity in larval zebrafish (Danio rerio). Sci Total Environ. 2023;882. | Map 2 | Yes |
| (6) Helm PA, Raby M, Kleywegt S, Sorichetti RJ, Arabian G, Smith D, et al. Assessment of tire-additive transformation product 6PPD-quinone in urban-impacted watersheds. ACS ES&T Wat. 2024:11. | Map 1 | Yes |
| (7) Johannessen C, Helm P, Metcalfe CD. Detection of selected tire wear compounds in urban receiving waters. Environ Pollut. 2021;287:117659. | Map 1 | Yes |
| (8) King MD, Rodgers TFM, Sharma G, Reger S, Liao XJ, Ross ARS, et al. Tracking 6PPD-Quinone Dynamics in a Coho Salmon-Bearing Stream Following Rain Reveals Elevated Concentrations for Multihour Periods During High Flow. Environmental Science & Technology Letters. 2025:6. | Map 1 | Yes |
| (9) Li JA, Xu JL, Jiang XD. Urban runoff mortality syndrome in zooplankton caused by tire wear particles. Environ Pollut. 2023;329:8. | Map 2 | Yes |
| (10) Li ZM, Jeong H, Kannan K. Occurrence and Distribution of 1,3-Diphenylguanidine, Benzotriazole, Benzothiazole, N-(1,3-Dimethylbutyl)-N′-phenyl-p-phenylenediamine, and Their Derivatives in Surface Water, Drinking Water, Stormwater Runoff, and Rainwater from New York State, USA. ACS ES&T Wat. 2025:11. | Map 1 | Yes |
| (11) Liu YH, Mei YX, Wang JY, Chen SS, Chen JL, Li N, et al. Precipitation contributes to alleviating pollution of rubber-derived chemicals in receiving watersheds: combining confluent stormwater runoff from different functional areas. Water Res. 2024;264:9. | Map 1 | Yes |
| (12) Liu ZQ, Feng YX, Sun WH, Wang BY, Shi CL, Ran RX, et al. Environmental concentrations of 6PPD and 6PPD-quinone induce hepatic lipid metabolism disorders in male black-spotted frogs. J Hazard Mater. 2024;480:11. | Map 2 | Yes |
| (13) Montgomery D, Ji XW, Cantin J, Philibert D, Foster G, Selinger S, et al. Interspecies differences in 6PPD-quinone toxicity across seven fish species: metabolite identification and semiquantification. Environ Sci Technol. 2023;57(50):21071-9. | Map 2 | Yes |
| (14) Philibert D, Stanton RS, Tang C, Stock NL, Benfey T, Pirrung M, de Jourdan B. The lethal and sublethal impacts of two tire rubber-derived chemicals on brook trout (*Salvelinus fontinalis*) fry and fingerlings. Chemosphere. 2024;360:142319. | Map 2 | Yes |
| (15) Prosser RS, Parrott JL, Galicia M, Shires K, Sullivan C, Toito J, et al. Toxicity of sediment-associated substituted phenylamine antioxidants on the early life stages of *Pimephales promelas* and a characterization of effects on freshwater organisms. Environ Toxicol Chem. 2017;36(10):2730-8. | Map 2 | Yes |
| (16) Prosser RS, Salole J, Hang S. Toxicity of 6PPD-quinone to four freshwater invertebrate species. Environ Pollut. 2023;337:6. | Map 1 and Map 2 | Yes |
| (17) Rauert C, Charlton N, Okoffo ED, Stanton RS, Agua AR, Pirrung MC, Thomas KV. Concentrations of tire additive chemicals and tire road wear particles in an Australian urban tributary. Environ Sci Technol. 2022;56(4):2421-31. | Map 1 | Yes |
| (18) Seiwert B, Nihemaiti M, Troussier M, Weyrauch S, Reemtsma T. Abiotic oxidative transformation of 6-PPD and 6-PPD quinone from tires and occurrence of their products in snow from urban roads and in municipal wastewater. Water Res. 2022;212:8. | Map 1 | Yes |
| (19) Selinger SJ, Montgomery D, Wiseman S, Hecker M, Weber L, Brinkmann M, Janz D. Acute cardiorespiratory effects of 6PPD-quinone on juvenile rainbow trout (*Oncorhynchus mykiss*) and arctic char (*Salvelinus alpinus*). Aquat Toxicol. 2025;280:11. | Map 2 | Yes |
| (20) Shankar P, Dalsky EM, Salzer JE, Lane RF, Hammond S, Batts WN, et al. Evaluation of 6PPD-Quinone Lethal Toxicity and Sublethal Effects on Disease Resistance and Swimming Performance in Coastal Cutthroat Trout (*Oncorhynchus clarkii clarkii*). Environ Sci Technol. 2025:10. | Map 2 | Yes |
| (21) Shi RY, Bao YY, Liu WT, Liu JZ, Wang XS, Shi XW, et al. Tire Rubber Antioxidant 6PPD and 6PPD-quinone Disrupt the Energy Supply and Lipid Metabolism of Earthworms. Environ Sci Technol. 2025:11. | Map 2 | Yes |
| (22) Tian ZY, Zhao HQ, Peter KT, Gonzalez M, Wetzel J, Wu C, et al. A ubiquitous tire rubber-derived chemical induces acute mortality in coho salmon. Science. 2021;371(6525):185-9. | Map 1 and 2 | Yes |
| (23) Wang W, Chen Y, Fang JC, Zhang F, Qu GB, Cai ZW. Toxicity of substituted p-phenylenediamine antioxidants and their derived novel quinones on aquatic bacterium: acute effects and mechanistic insights. J Hazard Mater. 2024;469:12. | Map 2 | Yes |
| (24) Wei LN, Wu NN, Xu R, Liu S, Li HX, Lin L, et al. First evidence of the bioaccumulation and trophic transfer of tire additives and their transformation products in an estuarine food web. Environ Sci Technol. 2024;58(14):6370-80. | Map 1 | Yes |
| (25) Wu W, Xu Q, Li JH, Wang Z, Li G. The spatio-temporal accumulation of 6 PPD-Q in greenbelt soils and its effects on soil microbial communities. Environ Pollut. 2024;358:8. | Map 1 | Yes |
| (26) Xie LN, Yu J, Nair P, Sun JX, Barrett H, Meek O, et al. Structurally selective ozonolysis of p-phenylenediamines and toxicity in coho salmon and rainbow trout. Environ Sci Technol. 2024:10. | Map 2 | Yes |
| (27) Zeng JJ, Li Y, Zhang Y, Dai SH, Wang JF, Zeng W, et al. Enantioselective accumulation and trophodynamics of p-phenylenediamine antioxidants and their quinones in the mangrove ecosystem. Environ Pollut. 2025;381:8. | Map 1 | Yes |
| (28) Zeng LX, Li Y, Sun YX, Liu LY, Shen MJ, Du BB. Widespread occurrence and transport of p-Phenylenediamines and their quinones in sediments across urban rivers, estuaries, coasts, and deep-sea regions. Environ Sci Technol. 2023:11. | Map 1 | Yes |
| (29) Zhang SY, Gan XF, Shen BG, Jiang J, Shen HM, Lei YH, et al. 6PPD and its metabolite 6PPDQ induce different developmental toxicities and phenotypes in embryonic zebrafish. J Hazard Mater. 2023;455:12. | Map 2 | Yes |
| (30) Zhu JQ, Guo RY, Ren FF, Jiang ST, Jin HB. Occurrence and partitioning of p-phenylenediamine antioxidants and their quinone derivatives in water and sediment. Sci Total Environ. 2024;914:7. | Map 1 | Yes |

**Table S5.** Summary of Web of Science (All Collections) search conducted on July 28 and 29, 2025. The results included preprint citations, all document types, and were refined to English language only.

| **Search terms included by category in Table S2 & S3** | **Number of search results** | **Benchmark articles return rate (%; 30 benchmark articles total)** | **Date of search** |
| --- | --- | --- | --- |
| All (acronym + identifier + chemical name + trade name + unknown) | 1,519,804 | 100% | July 28, 2025 |
| Acronyms | 812 | 100% | July 28, 2025 |
| Chemical names | 1,151 | 87%  (#3, 4, 23, and 27 not captured) | July 28, 2025 |
| Identifiers | 1,509,926 | 0% | July 28, 2025 |
| Trade names | 7,987 | 0% | July 28, 2025 |
| Acronyms + chemical names | 1,560 | 100% | July 28, 2025 |
| Acronyms + trade names | 8,796 | 100% | July 28, 2025 |
| Acronym + chemical name + trade name | 10,039 | 100% | July 28, 2025 |
| Identifiers (“CI” omitted) | 5 | 0% | July 29, 2025 |
| All (acronym + identifier (“CI” omitted) + chemical name + trade name + unknown) | 10,042 | 100% | July 29, 2025 |

**Table S6.** Summary of Web of Science (All Collections) search conducted on July 29, 2025 and updated on August 5, 2025. The results included preprint citations, all document types, and were refined to English language only.

| **Trade name in search** | **Number of search results** | **Number of results by document type:** | | | | | | | | | | | | | | | | | | | | |
| --- | --- | --- | --- | --- | --- | --- | --- | --- | --- | --- | --- | --- | --- | --- | --- | --- | --- | --- | --- | --- | --- | --- |
|  |  | Articles | Other | Meeting | Data set | Dissertation thesis | Patent | Abstract | Review article | Awarded grant | Clinical trial | Preprint | Letter | Early access | Editorial material | Data study | Book | Case report | Correction | Retracted publication | Reference material | Unspecified |
| "Accinox ZC" | 0 | 0 | 0 | 0 | 0 | 0 | 0 | 0 | 0 | 0 | 0 | 0 | 0 | 0 | 0 | 0 | 0 | 0 | 0 | 0 | 0 | 0 |
| ["Akrochem antiozonant pd-2"](https://can01.safelinks.protection.outlook.com/?url=https%3A%2F%2Fwww.ncbi.nlm.nih.gov%2Fpcsubstance%2F%3Fterm%3D%2522Akrochem%2520antiozonant%2520pd-2%2522%255BCompleteSynonym%255D%2520AND%252013101%255BStandardizedCID%255D&data=05%7C02%7Ck.seabrook%40queensu.ca%7Caf95b968c1d0459ac72a08ddd4fed5c8%7Cd61ecb3b38b142d582c4efb2838b925c%7C1%7C0%7C638900913168689284%7CUnknown%7CTWFpbGZsb3d8eyJFbXB0eU1hcGkiOnRydWUsIlYiOiIwLjAuMDAwMCIsIlAiOiJXaW4zMiIsIkFOIjoiTWFpbCIsIldUIjoyfQ%3D%3D%7C0%7C%7C%7C&sdata=StXIkmQ34nv36nG4ErGa%2Bzj%2FT6%2BPGKiTMzoVtW3O42g%3D&reserved=0) | 0 | 0 | 0 | 0 | 0 | 0 | 0 | 0 | 0 | 0 | 0 | 0 | 0 | 0 | 0 | 0 | 0 | 0 | 0 | 0 | 0 | 0 |
| ["Antage 6C"](https://can01.safelinks.protection.outlook.com/?url=https%3A%2F%2Fwww.ncbi.nlm.nih.gov%2Fpcsubstance%2F%3Fterm%3D%2522Antage%25206C%2522%255BCompleteSynonym%255D%2520AND%252013101%255BStandardizedCID%255D&data=05%7C02%7Ck.seabrook%40queensu.ca%7Caf95b968c1d0459ac72a08ddd4fed5c8%7Cd61ecb3b38b142d582c4efb2838b925c%7C1%7C0%7C638900913168706532%7CUnknown%7CTWFpbGZsb3d8eyJFbXB0eU1hcGkiOnRydWUsIlYiOiIwLjAuMDAwMCIsIlAiOiJXaW4zMiIsIkFOIjoiTWFpbCIsIldUIjoyfQ%3D%3D%7C0%7C%7C%7C&sdata=yM90GG82lKMy362UD3NSIDJnxAV8JwfxlJTxCopiNSw%3D&reserved=0) | 1 | 0 | 0 | 0 | 0 | 0 | 1 | 0 | 0 | 0 | 0 | 0 | 0 | 0 | 0 | 0 | 0 | 0 | 0 | 0 | 0 | 0 |
| "Antigene 6C" | 0 | 0 | 0 | 0 | 0 | 0 | 0 | 0 | 0 | 0 | 0 | 0 | 0 | 0 | 0 | 0 | 0 | 0 | 0 | 0 | 0 | 0 |
| ["Antioxidant 4020"](https://can01.safelinks.protection.outlook.com/?url=https%3A%2F%2Fwww.ncbi.nlm.nih.gov%2Fpcsubstance%2F%3Fterm%3D%2522Antioxidant%25204020%2522%255BCompleteSynonym%255D%2520AND%252013101%255BStandardizedCID%255D&data=05%7C02%7Ck.seabrook%40queensu.ca%7Caf95b968c1d0459ac72a08ddd4fed5c8%7Cd61ecb3b38b142d582c4efb2838b925c%7C1%7C0%7C638900913168716674%7CUnknown%7CTWFpbGZsb3d8eyJFbXB0eU1hcGkiOnRydWUsIlYiOiIwLjAuMDAwMCIsIlAiOiJXaW4zMiIsIkFOIjoiTWFpbCIsIldUIjoyfQ%3D%3D%7C0%7C%7C%7C&sdata=opItxwha54IzoZxhirTx07aLwxjpR2ntsqk4wOVU3Jo%3D&reserved=0) | 314 | 8 | 0 | 1 | 0 | 0 | 306 | 0 | 0 | 0 | 0 | 0 | 0 | 0 | 0 | 0 | 0 | 0 | 0 | 0 | 0 | 0 |
| "Antioxidant 6C" | 3 | 0 | 0 | 0 | 0 | 0 | 3 | 0 | 0 | 0 | 0 | 0 | 0 | 0 | 0 | 0 | 0 | 0 | 0 | 0 | 0 | 0 |
| ["Antioxidant CD 13"](https://can01.safelinks.protection.outlook.com/?url=https%3A%2F%2Fwww.ncbi.nlm.nih.gov%2Fpcsubstance%2F%3Fterm%3D%2522Antioxidant%2520CD%252013%2522%255BCompleteSynonym%255D%2520AND%252013101%255BStandardizedCID%255D&data=05%7C02%7Ck.seabrook%40queensu.ca%7Caf95b968c1d0459ac72a08ddd4fed5c8%7Cd61ecb3b38b142d582c4efb2838b925c%7C1%7C0%7C638900913168740446%7CUnknown%7CTWFpbGZsb3d8eyJFbXB0eU1hcGkiOnRydWUsIlYiOiIwLjAuMDAwMCIsIlAiOiJXaW4zMiIsIkFOIjoiTWFpbCIsIldUIjoyfQ%3D%3D%7C0%7C%7C%7C&sdata=j79%2FGynyh3EztKJeoAjVppZPkPrqjPybF3sTJaSEi5I%3D&reserved=0) | 0 | 0 | 0 | 0 | 0 | 0 | 0 | 0 | 0 | 0 | 0 | 0 | 0 | 0 | 0 | 0 | 0 | 0 | 0 | 0 | 0 | 0 |
| ["Antioxidant cd"](https://can01.safelinks.protection.outlook.com/?url=https%3A%2F%2Fwww.ncbi.nlm.nih.gov%2Fpcsubstance%2F%3Fterm%3D%2522Antioxidant%2520cd%2522%255BCompleteSynonym%255D%2520AND%252013101%255BStandardizedCID%255D&data=05%7C02%7Ck.seabrook%40queensu.ca%7Caf95b968c1d0459ac72a08ddd4fed5c8%7Cd61ecb3b38b142d582c4efb2838b925c%7C1%7C0%7C638900913168752266%7CUnknown%7CTWFpbGZsb3d8eyJFbXB0eU1hcGkiOnRydWUsIlYiOiIwLjAuMDAwMCIsIlAiOiJXaW4zMiIsIkFOIjoiTWFpbCIsIldUIjoyfQ%3D%3D%7C0%7C%7C%7C&sdata=dK%2Fj1%2F%2BfxwbJi1NehQb0VoxdfonrJ4X1zQ4V1zAZAso%3D&reserved=0) | 6 | 3 | 1 | 0 | 0 | 0 | 3 | 0 | 0 | 0 | 0 | 0 | 0 | 0 | 0 | 0 | 0 | 0 | 0 | 0 | 0 | 0 |
| "Antioxidant PD 2" | 0 | 0 | 0 | 0 | 0 | 0 | 0 | 0 | 0 | 0 | 0 | 0 | 0 | 0 | 0 | 0 | 0 | 0 | 0 | 0 | 0 | 0 |
| ["Antozite 67"](https://can01.safelinks.protection.outlook.com/?url=https%3A%2F%2Fwww.ncbi.nlm.nih.gov%2Fpcsubstance%2F%3Fterm%3D%2522Antozite%252067%2522%255BCompleteSynonym%255D%2520AND%252013101%255BStandardizedCID%255D&data=05%7C02%7Ck.seabrook%40queensu.ca%7Caf95b968c1d0459ac72a08ddd4fed5c8%7Cd61ecb3b38b142d582c4efb2838b925c%7C1%7C0%7C638900913168763233%7CUnknown%7CTWFpbGZsb3d8eyJFbXB0eU1hcGkiOnRydWUsIlYiOiIwLjAuMDAwMCIsIlAiOiJXaW4zMiIsIkFOIjoiTWFpbCIsIldUIjoyfQ%3D%3D%7C0%7C%7C%7C&sdata=S%2FXz%2FnvoR%2Bg2822mMvj014Xc1TDD9iFjI1CNN1MqTdk%3D&reserved=0) | 0 | 0 | 0 | 0 | 0 | 0 | 0 | 0 | 0 | 0 | 0 | 0 | 0 | 0 | 0 | 0 | 0 | 0 | 0 | 0 | 0 | 0 |
| ["Antozite 67F"](https://can01.safelinks.protection.outlook.com/?url=https%3A%2F%2Fwww.ncbi.nlm.nih.gov%2Fpcsubstance%2F%3Fterm%3D%2522Antozite%252067F%2522%255BCompleteSynonym%255D%2520AND%252013101%255BStandardizedCID%255D&data=05%7C02%7Ck.seabrook%40queensu.ca%7Caf95b968c1d0459ac72a08ddd4fed5c8%7Cd61ecb3b38b142d582c4efb2838b925c%7C1%7C0%7C638900913168773957%7CUnknown%7CTWFpbGZsb3d8eyJFbXB0eU1hcGkiOnRydWUsIlYiOiIwLjAuMDAwMCIsIlAiOiJXaW4zMiIsIkFOIjoiTWFpbCIsIldUIjoyfQ%3D%3D%7C0%7C%7C%7C&sdata=X%2BevhQwXNHol%2FHl82AzTdVdf88JuMiJJbgCgeX85EpE%3D&reserved=0) | 0 | 0 | 0 | 0 | 0 | 0 | 0 | 0 | 0 | 0 | 0 | 0 | 0 | 0 | 0 | 0 | 0 | 0 | 0 | 0 | 0 | 0 |
| ["Diafen 13"](https://can01.safelinks.protection.outlook.com/?url=https%3A%2F%2Fwww.ncbi.nlm.nih.gov%2Fpcsubstance%2F%3Fterm%3D%2522Diafen%252013%2522%255BCompleteSynonym%255D%2520AND%252013101%255BStandardizedCID%255D&data=05%7C02%7Ck.seabrook%40queensu.ca%7Caf95b968c1d0459ac72a08ddd4fed5c8%7Cd61ecb3b38b142d582c4efb2838b925c%7C1%7C0%7C638900913168784442%7CUnknown%7CTWFpbGZsb3d8eyJFbXB0eU1hcGkiOnRydWUsIlYiOiIwLjAuMDAwMCIsIlAiOiJXaW4zMiIsIkFOIjoiTWFpbCIsIldUIjoyfQ%3D%3D%7C0%7C%7C%7C&sdata=uAQyo6puXTN64fWc7gShfYnmsaHs6apYwCgepYWK%2FbY%3D&reserved=0) | 0 | 0 | 0 | 0 | 0 | 0 | 0 | 0 | 0 | 0 | 0 | 0 | 0 | 0 | 0 | 0 | 0 | 0 | 0 | 0 | 0 | 0 |
| ["Diafen FDMB"](https://can01.safelinks.protection.outlook.com/?url=https%3A%2F%2Fwww.ncbi.nlm.nih.gov%2Fpcsubstance%2F%3Fterm%3D%2522Diafen%2520FDMB%2522%255BCompleteSynonym%255D%2520AND%252013101%255BStandardizedCID%255D&data=05%7C02%7Ck.seabrook%40queensu.ca%7Caf95b968c1d0459ac72a08ddd4fed5c8%7Cd61ecb3b38b142d582c4efb2838b925c%7C1%7C0%7C638900913168794981%7CUnknown%7CTWFpbGZsb3d8eyJFbXB0eU1hcGkiOnRydWUsIlYiOiIwLjAuMDAwMCIsIlAiOiJXaW4zMiIsIkFOIjoiTWFpbCIsIldUIjoyfQ%3D%3D%7C0%7C%7C%7C&sdata=eB28Bkwgt%2BOw5R6lKr1cbFTwTLCbRkcqScGUYQe6jyM%3D&reserved=0) | 0 | 0 | 0 | 0 | 0 | 0 | 0 | 0 | 0 | 0 | 0 | 0 | 0 | 0 | 0 | 0 | 0 | 0 | 0 | 0 | 0 | 0 |
| ["Dusantox 6PPD"](https://can01.safelinks.protection.outlook.com/?url=https%3A%2F%2Fwww.ncbi.nlm.nih.gov%2Fpcsubstance%2F%3Fterm%3D%2522Dusantox%25206PPD%2522%255BCompleteSynonym%255D%2520AND%252013101%255BStandardizedCID%255D&data=05%7C02%7Ck.seabrook%40queensu.ca%7Caf95b968c1d0459ac72a08ddd4fed5c8%7Cd61ecb3b38b142d582c4efb2838b925c%7C1%7C0%7C638900913168805067%7CUnknown%7CTWFpbGZsb3d8eyJFbXB0eU1hcGkiOnRydWUsIlYiOiIwLjAuMDAwMCIsIlAiOiJXaW4zMiIsIkFOIjoiTWFpbCIsIldUIjoyfQ%3D%3D%7C0%7C%7C%7C&sdata=M1Lzr63uxR5gb%2BYr2gQd%2Bu%2Fnvq2mlOKn2b%2BM2etihdc%3D&reserved=0) | 1 | 0 | 0 | 0 | 0 | 0 | 1 | 0 | 0 | 0 | 0 | 0 | 0 | 0 | 0 | 0 | 0 | 0 | 0 | 0 | 0 | 0 |
| ["Flexzone 7F"](https://can01.safelinks.protection.outlook.com/?url=https%3A%2F%2Fwww.ncbi.nlm.nih.gov%2Fpcsubstance%2F%3Fterm%3D%2522Flexzone%25207F%2522%255BCompleteSynonym%255D%2520AND%252013101%255BStandardizedCID%255D&data=05%7C02%7Ck.seabrook%40queensu.ca%7Caf95b968c1d0459ac72a08ddd4fed5c8%7Cd61ecb3b38b142d582c4efb2838b925c%7C1%7C0%7C638900913168815383%7CUnknown%7CTWFpbGZsb3d8eyJFbXB0eU1hcGkiOnRydWUsIlYiOiIwLjAuMDAwMCIsIlAiOiJXaW4zMiIsIkFOIjoiTWFpbCIsIldUIjoyfQ%3D%3D%7C0%7C%7C%7C&sdata=Dg3PQyN4TU5w1j3pxjW7fOATTg222MlO%2FMj4cjgZ2rw%3D&reserved=0) | 0 | 0 | 0 | 0 | 0 | 0 | 0 | 0 | 0 | 0 | 0 | 0 | 0 | 0 | 0 | 0 | 0 | 0 | 0 | 0 | 0 | 0 |
| ["Flexzone 7L"](https://can01.safelinks.protection.outlook.com/?url=https%3A%2F%2Fwww.ncbi.nlm.nih.gov%2Fpcsubstance%2F%3Fterm%3D%2522Flexzone%25207L%2522%255BCompleteSynonym%255D%2520AND%252013101%255BStandardizedCID%255D&data=05%7C02%7Ck.seabrook%40queensu.ca%7Caf95b968c1d0459ac72a08ddd4fed5c8%7Cd61ecb3b38b142d582c4efb2838b925c%7C1%7C0%7C638900913168825360%7CUnknown%7CTWFpbGZsb3d8eyJFbXB0eU1hcGkiOnRydWUsIlYiOiIwLjAuMDAwMCIsIlAiOiJXaW4zMiIsIkFOIjoiTWFpbCIsIldUIjoyfQ%3D%3D%7C0%7C%7C%7C&sdata=ObKHnVKRKVstwmbP%2F%2BUudjRdVlyucc%2B6cC4vfM2hd3U%3D&reserved=0) | 0 | 0 | 0 | 0 | 0 | 0 | 0 | 0 | 0 | 0 | 0 | 0 | 0 | 0 | 0 | 0 | 0 | 0 | 0 | 0 | 0 | 0 |
| "Flexzone 7P" | 1 | 1 | 1 | 0 | 0 | 0 | 0 | 0 | 0 | 0 | 0 | 0 | 0 | 0 | 0 | 0 | 0 | 0 | 0 | 0 | 0 | 0 |
| ["Forte 6C"](https://can01.safelinks.protection.outlook.com/?url=https%3A%2F%2Fwww.ncbi.nlm.nih.gov%2Fpcsubstance%2F%3Fterm%3D%2522Forte%25206C%2522%255BCompleteSynonym%255D%2520AND%252013101%255BStandardizedCID%255D&data=05%7C02%7Ck.seabrook%40queensu.ca%7Caf95b968c1d0459ac72a08ddd4fed5c8%7Cd61ecb3b38b142d582c4efb2838b925c%7C1%7C0%7C638900913168835385%7CUnknown%7CTWFpbGZsb3d8eyJFbXB0eU1hcGkiOnRydWUsIlYiOiIwLjAuMDAwMCIsIlAiOiJXaW4zMiIsIkFOIjoiTWFpbCIsIldUIjoyfQ%3D%3D%7C0%7C%7C%7C&sdata=6tWzQ8lpPhvfLfs7R%2BYJFJAoROuwM1agZETP1i7UFfU%3D&reserved=0) | 0 | 0 | 0 | 0 | 0 | 0 | 0 | 0 | 0 | 0 | 0 | 0 | 0 | 0 | 0 | 0 | 0 | 0 | 0 | 0 | 0 | 0 |
| "Kumanox 13" | 2 | 0 | 0 | 0 | 0 | 0 | 2 | 0 | 0 | 0 | 0 | 0 | 0 | 0 | 0 | 0 | 0 | 0 | 0 | 0 | 0 | 0 |
| "Kumanox 13C" | 0 | 0 | 0 | 0 | 0 | 0 | 0 | 0 | 0 | 0 | 0 | 0 | 0 | 0 | 0 | 0 | 0 | 0 | 0 | 0 | 0 | 0 |
| "Luvomaxx 6PPD" | 0 | 0 | 0 | 0 | 0 | 0 | 0 | 0 | 0 | 0 | 0 | 0 | 0 | 0 | 0 | 0 | 0 | 0 | 0 | 0 | 0 | 0 |
| "Nocceler 6C" | 0 | 0 | 0 | 0 | 0 | 0 | 0 | 0 | 0 | 0 | 0 | 0 | 0 | 0 | 0 | 0 | 0 | 0 | 0 | 0 | 0 | 0 |
| ["Nocrac 6C"](https://can01.safelinks.protection.outlook.com/?url=https%3A%2F%2Fwww.ncbi.nlm.nih.gov%2Fpcsubstance%2F%3Fterm%3D%2522Nocrac%25206C%2522%255BCompleteSynonym%255D%2520AND%252013101%255BStandardizedCID%255D&data=05%7C02%7Ck.seabrook%40queensu.ca%7Caf95b968c1d0459ac72a08ddd4fed5c8%7Cd61ecb3b38b142d582c4efb2838b925c%7C1%7C0%7C638900913168845516%7CUnknown%7CTWFpbGZsb3d8eyJFbXB0eU1hcGkiOnRydWUsIlYiOiIwLjAuMDAwMCIsIlAiOiJXaW4zMiIsIkFOIjoiTWFpbCIsIldUIjoyfQ%3D%3D%7C0%7C%7C%7C&sdata=qVq65HALuDMraNShlKg2G75dKQoBiCw7QVtcaGIrXOA%3D&reserved=0) | 0 | 0 | 0 | 0 | 0 | 0 | 0 | 0 | 0 | 0 | 0 | 0 | 0 | 0 | 0 | 0 | 0 | 0 | 0 | 0 | 0 | 0 |
| ["Nocrane 6C"](https://can01.safelinks.protection.outlook.com/?url=https%3A%2F%2Fwww.ncbi.nlm.nih.gov%2Fpcsubstance%2F%3Fterm%3D%2522Nocrane%25206C%2522%255BCompleteSynonym%255D%2520AND%252013101%255BStandardizedCID%255D&data=05%7C02%7Ck.seabrook%40queensu.ca%7Caf95b968c1d0459ac72a08ddd4fed5c8%7Cd61ecb3b38b142d582c4efb2838b925c%7C1%7C0%7C638900913168856146%7CUnknown%7CTWFpbGZsb3d8eyJFbXB0eU1hcGkiOnRydWUsIlYiOiIwLjAuMDAwMCIsIlAiOiJXaW4zMiIsIkFOIjoiTWFpbCIsIldUIjoyfQ%3D%3D%7C0%7C%7C%7C&sdata=Yi05PMiFS4v%2FIdySLJZMEW%2Bbs7AlGX2lrN4CDyVFsTc%3D&reserved=0) | 0 | 0 | 0 | 0 | 0 | 0 | 0 | 0 | 0 | 0 | 0 | 0 | 0 | 0 | 0 | 0 | 0 | 0 | 0 | 0 | 0 | 0 |
| ["Nocrane 7 L"](https://can01.safelinks.protection.outlook.com/?url=https%3A%2F%2Fwww.ncbi.nlm.nih.gov%2Fpcsubstance%2F%3Fterm%3D%2522Nocrane%25207%2520L%2522%255BCompleteSynonym%255D%2520AND%252013101%255BStandardizedCID%255D&data=05%7C02%7Ck.seabrook%40queensu.ca%7Caf95b968c1d0459ac72a08ddd4fed5c8%7Cd61ecb3b38b142d582c4efb2838b925c%7C1%7C0%7C638900913168867694%7CUnknown%7CTWFpbGZsb3d8eyJFbXB0eU1hcGkiOnRydWUsIlYiOiIwLjAuMDAwMCIsIlAiOiJXaW4zMiIsIkFOIjoiTWFpbCIsIldUIjoyfQ%3D%3D%7C0%7C%7C%7C&sdata=hwkvZGKWGGcTdDXZ5jT8jubP3MXonUHaXIzYVlbhL7E%3D&reserved=0) | 0 | 0 | 0 | 0 | 0 | 0 | 0 | 0 | 0 | 0 | 0 | 0 | 0 | 0 | 0 | 0 | 0 | 0 | 0 | 0 | 0 | 0 |
| ["Ozonon 6C"](https://can01.safelinks.protection.outlook.com/?url=https%3A%2F%2Fwww.ncbi.nlm.nih.gov%2Fpcsubstance%2F%3Fterm%3D%2522Ozonon%25206C%2522%255BCompleteSynonym%255D%2520AND%252013101%255BStandardizedCID%255D&data=05%7C02%7Ck.seabrook%40queensu.ca%7Caf95b968c1d0459ac72a08ddd4fed5c8%7Cd61ecb3b38b142d582c4efb2838b925c%7C1%7C0%7C638900913168880747%7CUnknown%7CTWFpbGZsb3d8eyJFbXB0eU1hcGkiOnRydWUsIlYiOiIwLjAuMDAwMCIsIlAiOiJXaW4zMiIsIkFOIjoiTWFpbCIsIldUIjoyfQ%3D%3D%7C0%7C%7C%7C&sdata=QLgbkOIz0vbJU%2B%2BsOnB3Ymp5IWnYyCGS0UX7wa06av0%3D&reserved=0) | 0 | 0 | 0 | 0 | 0 | 0 | 0 | 0 | 0 | 0 | 0 | 0 | 0 | 0 | 0 | 0 | 0 | 0 | 0 | 0 | 0 | 0 |
| ["Ozonone 6C"](https://can01.safelinks.protection.outlook.com/?url=https%3A%2F%2Fwww.ncbi.nlm.nih.gov%2Fpcsubstance%2F%3Fterm%3D%2522Ozonone%25206C%2522%255BCompleteSynonym%255D%2520AND%252013101%255BStandardizedCID%255D&data=05%7C02%7Ck.seabrook%40queensu.ca%7Caf95b968c1d0459ac72a08ddd4fed5c8%7Cd61ecb3b38b142d582c4efb2838b925c%7C1%7C0%7C638900913168895788%7CUnknown%7CTWFpbGZsb3d8eyJFbXB0eU1hcGkiOnRydWUsIlYiOiIwLjAuMDAwMCIsIlAiOiJXaW4zMiIsIkFOIjoiTWFpbCIsIldUIjoyfQ%3D%3D%7C0%7C%7C%7C&sdata=soCnsiGLuJhvw8pjCjGcrSRkaGVGrtVspr0N5ZmsNvw%3D&reserved=0) | 0 | 0 | 0 | 0 | 0 | 0 | 0 | 0 | 0 | 0 | 0 | 0 | 0 | 0 | 0 | 0 | 0 | 0 | 0 | 0 | 0 | 0 |
| "PD 2" | 7,637 | 6,106 | 1,513 | 503 | 385 | 379 | 298 | 201 | 151 | 108 | 66 | 47 | 22 | 17 | 16 | 13 | 12 | 10 | 5 | 4 | 2 | 2 |
| ["Permanax 120"](https://can01.safelinks.protection.outlook.com/?url=https%3A%2F%2Fwww.ncbi.nlm.nih.gov%2Fpcsubstance%2F%3Fterm%3D%2522Permanax%2520120%2522%255BCompleteSynonym%255D%2520AND%252013101%255BStandardizedCID%255D&data=05%7C02%7Ck.seabrook%40queensu.ca%7Caf95b968c1d0459ac72a08ddd4fed5c8%7Cd61ecb3b38b142d582c4efb2838b925c%7C1%7C0%7C638900913168907640%7CUnknown%7CTWFpbGZsb3d8eyJFbXB0eU1hcGkiOnRydWUsIlYiOiIwLjAuMDAwMCIsIlAiOiJXaW4zMiIsIkFOIjoiTWFpbCIsIldUIjoyfQ%3D%3D%7C0%7C%7C%7C&sdata=kUnK58nYMBrK33hILtZk99R0Xe6hFsZNw2hqF0NKhqA%3D&reserved=0) | 0 | 0 | 0 | 0 | 0 | 0 | 0 | 0 | 0 | 0 | 0 | 0 | 0 | 0 | 0 | 0 | 0 | 0 | 0 | 0 | 0 | 0 |
| ["Permanax 6PPD"](https://can01.safelinks.protection.outlook.com/?url=https%3A%2F%2Fwww.ncbi.nlm.nih.gov%2Fpcsubstance%2F%3Fterm%3D%2522Permanax%25206PPD%2522%255BCompleteSynonym%255D%2520AND%252013101%255BStandardizedCID%255D&data=05%7C02%7Ck.seabrook%40queensu.ca%7Caf95b968c1d0459ac72a08ddd4fed5c8%7Cd61ecb3b38b142d582c4efb2838b925c%7C1%7C0%7C638900913168919781%7CUnknown%7CTWFpbGZsb3d8eyJFbXB0eU1hcGkiOnRydWUsIlYiOiIwLjAuMDAwMCIsIlAiOiJXaW4zMiIsIkFOIjoiTWFpbCIsIldUIjoyfQ%3D%3D%7C0%7C%7C%7C&sdata=R2vevgOh716ph4LCKiD7f85fB4k9NxfccbZFz6e1MbI%3D&reserved=0) | 0 | 0 | 0 | 0 | 0 | 0 | 0 | 0 | 0 | 0 | 0 | 0 | 0 | 0 | 0 | 0 | 0 | 0 | 0 | 0 | 0 | 0 |
| "Pilflex 13" | 0 | 0 | 0 | 0 | 0 | 0 | 0 | 0 | 0 | 0 | 0 | 0 | 0 | 0 | 0 | 0 | 0 | 0 | 0 | 0 | 0 | 0 |
| "Rubatan BF" | 0 | 0 | 0 | 0 | 0 | 0 | 0 | 0 | 0 | 0 | 0 | 0 | 0 | 0 | 0 | 0 | 0 | 0 | 0 | 0 | 0 | 0 |
| ["Santoflex 13"](https://can01.safelinks.protection.outlook.com/?url=https%3A%2F%2Fwww.ncbi.nlm.nih.gov%2Fpcsubstance%2F%3Fterm%3D%2522Santoflex%252013%2522%255BCompleteSynonym%255D%2520AND%252013101%255BStandardizedCID%255D&data=05%7C02%7Ck.seabrook%40queensu.ca%7Caf95b968c1d0459ac72a08ddd4fed5c8%7Cd61ecb3b38b142d582c4efb2838b925c%7C1%7C0%7C638900913168931027%7CUnknown%7CTWFpbGZsb3d8eyJFbXB0eU1hcGkiOnRydWUsIlYiOiIwLjAuMDAwMCIsIlAiOiJXaW4zMiIsIkFOIjoiTWFpbCIsIldUIjoyfQ%3D%3D%7C0%7C%7C%7C&sdata=%2BFYqrEAQOD0bkBgeHLUgVJaJAvsZNz%2BmxrBZOoUtZH8%3D&reserved=0) | 1 | 1 | 0 | 0 | 0 | 0 | 0 | 0 | 0 | 0 | 0 | 0 | 0 | 0 | 0 | 0 | 0 | 0 | 0 | 0 | 0 | 0 |
| ["Santoflex 13F"](https://can01.safelinks.protection.outlook.com/?url=https%3A%2F%2Fwww.ncbi.nlm.nih.gov%2Fpcsubstance%2F%3Fterm%3D%2522Santoflex%252013F%2522%255BCompleteSynonym%255D%2520AND%252013101%255BStandardizedCID%255D&data=05%7C02%7Ck.seabrook%40queensu.ca%7Caf95b968c1d0459ac72a08ddd4fed5c8%7Cd61ecb3b38b142d582c4efb2838b925c%7C1%7C0%7C638900913168941858%7CUnknown%7CTWFpbGZsb3d8eyJFbXB0eU1hcGkiOnRydWUsIlYiOiIwLjAuMDAwMCIsIlAiOiJXaW4zMiIsIkFOIjoiTWFpbCIsIldUIjoyfQ%3D%3D%7C0%7C%7C%7C&sdata=fQhWBcg6%2BIRxCTBl4hCzWs21ht3bT871yNoKUc26BLM%3D&reserved=0) | 0 | 0 | 0 | 0 | 0 | 0 | 0 | 0 | 0 | 0 | 0 | 0 | 0 | 0 | 0 | 0 | 0 | 0 | 0 | 0 | 0 | 0 |
| ["Santoflex 6PPD"](https://can01.safelinks.protection.outlook.com/?url=https%3A%2F%2Fwww.ncbi.nlm.nih.gov%2Fpcsubstance%2F%3Fterm%3D%2522Santoflex%25206PPD%2522%255BCompleteSynonym%255D%2520AND%252013101%255BStandardizedCID%255D&data=05%7C02%7Ck.seabrook%40queensu.ca%7Caf95b968c1d0459ac72a08ddd4fed5c8%7Cd61ecb3b38b142d582c4efb2838b925c%7C1%7C0%7C638900913168952464%7CUnknown%7CTWFpbGZsb3d8eyJFbXB0eU1hcGkiOnRydWUsIlYiOiIwLjAuMDAwMCIsIlAiOiJXaW4zMiIsIkFOIjoiTWFpbCIsIldUIjoyfQ%3D%3D%7C0%7C%7C%7C&sdata=GdsynhFBWv3trSx5KOVyqGvAlUnHlDiSHWQPBkj5Af0%3D&reserved=0) | 0 | 0 | 0 | 0 | 0 | 0 | 0 | 0 | 0 | 0 | 0 | 0 | 0 | 0 | 0 | 0 | 0 | 0 | 0 | 0 | 0 | 0 |
| "Stangard 6PPD" | 0 | 0 | 0 | 0 | 0 | 0 | 0 | 0 | 0 | 0 | 0 | 0 | 0 | 0 | 0 | 0 | 0 | 0 | 0 | 0 | 0 | 0 |
| "Sunsine 6PPD" | 0 | 0 | 0 | 0 | 0 | 0 | 0 | 0 | 0 | 0 | 0 | 0 | 0 | 0 | 0 | 0 | 0 | 0 | 0 | 0 | 0 | 0 |
| "UOP 562" | 0 | 0 | 0 | 0 | 0 | 0 | 0 | 0 | 0 | 0 | 0 | 0 | 0 | 0 | 0 | 0 | 0 | 0 | 0 | 0 | 0 | 0 |
| "UOP 588" | 0 | 0 | 0 | 0 | 0 | 0 | 0 | 0 | 0 | 0 | 0 | 0 | 0 | 0 | 0 | 0 | 0 | 0 | 0 | 0 | 0 | 0 |
| "Vulkanox 4020" | 1 | 1 | 0 | 0 | 0 | 0 | 0 | 0 | 0 | 0 | 0 | 0 | 0 | 0 | 0 | 0 | 0 | 0 | 0 | 0 | 0 | 0 |
| "Vulkanox 4020LG" | 0 | 0 | 0 | 0 | 0 | 0 | 0 | 0 | 0 | 0 | 0 | 0 | 0 | 0 | 0 | 0 | 0 | 0 | 0 | 0 | 0 | 0 |
| ["Wingstay 300"](https://can01.safelinks.protection.outlook.com/?url=https%3A%2F%2Fwww.ncbi.nlm.nih.gov%2Fpcsubstance%2F%3Fterm%3D%2522Wingstay%2520300%2522%255BCompleteSynonym%255D%2520AND%252013101%255BStandardizedCID%255D&data=05%7C02%7Ck.seabrook%40queensu.ca%7Caf95b968c1d0459ac72a08ddd4fed5c8%7Cd61ecb3b38b142d582c4efb2838b925c%7C1%7C0%7C638900913168963073%7CUnknown%7CTWFpbGZsb3d8eyJFbXB0eU1hcGkiOnRydWUsIlYiOiIwLjAuMDAwMCIsIlAiOiJXaW4zMiIsIkFOIjoiTWFpbCIsIldUIjoyfQ%3D%3D%7C0%7C%7C%7C&sdata=%2BM5nutXrz5FlUwAN42k7YEdozeDv5WZzgcUwUIbymGM%3D&reserved=0) | 0 | 0 | 0 | 0 | 0 | 0 | 0 | 0 | 0 | 0 | 0 | 0 | 0 | 0 | 0 | 0 | 0 | 0 | 0 | 0 | 0 | 0 |
| “PX-13” | 25 | 9 | 2 | 2 | 15 | 0 | 1 | 0 | 0 | 0 | 0 | 0 | 0 | 0 | 0 | 0 | 0 | 0 | 0 | 0 | 0 | 0 |

**Table S7.** Summary of Web of Science (All Collections) search for individual search terms conducted on August 04, 2025.

| **Search term** | **Search term category** | **Number of search results (including preprints, English language only)** | **Number of articles in search results** |
| --- | --- | --- | --- |
| “*p*-Phenylenediamine, *N*-(1,3-dimethylbutyl)-*N*′-phenyl- (7CI, 8CI)” | Chemical name | 0 | 0 |
| “CI” | Identifier | 1,513,374 | 1,247,681 |
| "Akrochem antiozonant pd-2" | Trade name | 0 | 0 |
| “PD 2” | Trade name | 7,637 | 6,106 |

**Table S8.** Review of title and abstract of “CI” articles to assess relevance to 6PPD/6PPDQ map protocols. The articles were sorted by relevance and the first 40 articles were reviewed.

| **Result # sorted by relevance** | **Topic** | **Relevance to 6PPD/6PPDQ map protocols** |
| --- | --- | --- |
| 1 | Mathematics, pseudo CI-filter | Not relevant |
| 2 | Genetics, R(ci) alleles in heterozygotes | Not relevant |
| 3 | Mathematics, cubic intuitionistic (CI) | Not relevant |
| 4 | Microbiology, C1-# strains | Not relevant |
| 5 | Medical, cardiac index (CI) | Not relevant |
| 6 | Medical, cochlear implant (CI) | Not relevant |
| 7 | Mathematics, pseudo CI-filter | Not relevant |
| 8 | Microbiology, Cyclic isomaltooligosaccharides (CIs), CI-# | Not relevant |
| 9 | Sustainability, Critical infrastructure (CI) | Not relevant |
| 10 | Physiology, Ci/Gli transcription factor and proteins | Not relevant |
| 11 | Medical, colonic ischaemia (CI) | Not relevant |
| 12 | Medical, cardiac index (CI) | Not relevant |
| 13 | Medical, cochlear implant (CI) | Not relevant |
| 14 | Medical, Inferior vena cava collapsibility index (IVC-CI) | Not relevant |
| 15 | Physics, CI-SDTQ | Not relevant |
| 16 | Japanese business, competitive intelligence (CI) | Not relevant |
| 17 | Medical, cochlear implant (CI) | Not relevant |
| 18 | Genetics, allele ci | Not relevant |
| 19 | Business, continuous improvement (CI) | Not relevant |
| 20 | Biology, Ciona intestinalis (Ci) | Not relevant |
| 21 | Medical, cochlear implant (CI) | Not relevant |
| 22 | Biology | Not relevant |
| 23 | Biology, milky mushroom C1-# strains | Not relevant |
| 24 | Medical, contrast-induced acute kidney injury (CI-AKI) | Not relevant |
| 25 | Physics, SAC-CI (symmetry-adapted-cluster configuration-interaction) | Not relevant |
| 26 | Medical, cochlear implant (CI) | Not relevant |
| 27 | Azo pigments, CI not in abstract | Not relevant |
| 28 | Genetics, Zic family zinc-finger protein Ci-macho! | Not relevant |
| 29 | Genetics, cubitus interruptus (ci) | Not relevant |
| 30 | Medical, cochlear implants (CI) | Not relevant |
| 31 | Science, confidence interval | Not relevant |
| 32 | Business, competitive intelligence (CI) | Not relevant |
| 33 | Medical, cochlear implantation (CI) | Not relevant |
| 34 | Medical, cochlear implant (CI) | Not relevant |
| 35 | Business, continuous improvement (CI) in supply chain | Not relevant |
| 36 | Medical, cardiac index (CI) | Not relevant |
| 37 | Medical, confidence interval | Not relevant |
| 38 | Medical, cerebral infarction (CI) | Not relevant |
| 39 | Physics, SAC CI calculations | Not relevant |
| 40 | Medical, cochlear implants (CIs) | Not relevant |

**Table S9.** Review of title and abstract of “PD 2” articles to assess relevance to 6PPD/6PPDQ map protocols. The articles were sorted by relevance and the first 40 articles were reviewed.

| **Result # sorted by relevance** | **Topic** | **Relevance to 6PPD/6PPDQ map protocols** |
| --- | --- | --- |
| 1 | Chemistry, palladium acetate complexes, Pd-2(OOCMe) (4) (+) , Pd-2(OOCMe) (3) (+) , Pd-2(OOCMe) (2) (+) , and Pd-2(OOCMe)(+) | Not relevant |
| 2 | Chemistry, dipalladium(I) cationic complex [Pd-2(mu-PBu2t)(mu-(PBu2H)-H-t)((PBu2H)-H-t)(2)]BF4 1 | Not relevant |
| 3 | Chemistry, Pd(II) p-tolylamide Pd(2,6-(Ph2PCH2)(2)C6H3)(NH(C6H4Me-p)) | Not relevant |
| 4 | Chemistry, Pd(OAc)(2) | Not relevant |
| 5 | Chemistry, Pd-2(1) complexes Pd2X2(dMPM)(2) | Not relevant |
| 6 | Chemistry, [Pd-2(edt)(2)(PPh(3))(2)] | Not relevant |
| 7 | Chemistry, [Pd-2(dmba)(2)Cl-2(mu-bpe)] | Not relevant |
| 8 | Chemistry, dipalladium(I) complex [Pd-2(CH3CN)(6)][BF4](2) | Not relevant |
| 9 | Chemistry, [Pd-2(C6F5)(4)(mu-OH)(mu-NHR)](2-) (R = C6H5, C6H4-Cl-p, C6H4F-p) | Not relevant |
| 10 | Chemistry, [Pd-2(mu-PBu(2)(t))(PR(3))(4)](+) (PR(3) = PCy(2)H, PMe(3)) | Not relevant |
| 11 | Chemistry, Pd(2+)ions | Not relevant |
| 12 | Chemistry, [Pd-2(C7H4NS2)(4)] | Not relevant |
| 13 | Chemistry, dinuclear cyclometallated complexes [{Pd [2-ClC6H3C(H) = NCH2CH2SMe] (Cl)}(2) {mu-Ph2P(CH2)(n)PPh2}] | Not relevant |
| 14 | Chemistry, Pd-2(4+) core, [Pd(eta(2)- dithio)](2)(mu-dppa)(mu-SCNMe2) | Not relevant |
| 15 | Chemistry, (Bu(4)N)(2)Pd(2)Br(6) | Not relevant |
| 16 | Chemistry, Pd-2[(C6H4)PPh2](2)[R'NC(H)NR'] | Not relevant |
| 17 | Chemistry, PD-2+ | Not relevant |
| 18 | Chemistry, Pd-2(III,III) complexes | Not relevant |
| 19 | Chemistry[Pd-2(hfac)(2)(mu-MeO)(2)] | Not relevant |
| 20 | Chemistry, [Pd-2(1-2H)(OAc)(2)] | Not relevant |
| 21 | Chemistry, dinuclear cyclopalladated compounds [Pd-2(mu -OAc)(2)(L-n)(2)] | Not relevant |
| 22 | Chemistry, PD(2)-1-AMINO-2-NAPHTHOL-4-SULPHONIC ACID | Not relevant |
| 23 | Chemistry, PD]2(MU-O2CME)2 AND [(MEC6H3C7H4NO)PD]2(MU-O2CME)2 | Not relevant |
| 24 | Chemistry, [Pd2R2(mu -X)(mu -dppm)(2)]PF6 | Not relevant |
| 25 | Chemistry, Pd(2)-P(2) 2.2290(10) | Not relevant |
| 26 | Chemistry, arylpalladium complex [Pd(2-C6H4CH2OSi-t-BuMe2)(PPh3)(2)I] | Not relevant |
| 27 | Chemistry, Complex cis-Pd(2,2'-bipyridine)(NO3)(2) | Not relevant |
| 28 | Chemistry, bimetallic palladium complex (BPB-Pd-2) | Not relevant |
| 29 | Chemistry, Pd(2+) | Not relevant |
| 30 | Chemistry, binuclear Pd(I) complex [Pd-2(mu-dppa)(2)(CH3CN)(2)][BF4](2) (1) | Not relevant |
| 31 | Chemistry, not highlighted in abstract | Not relevant |
| 32 | Chemistry, Pd-2(dppm)(2)Cl-2 (dppm = Ph(2)PCH2PPh(2)), [Pd-4(dppm)(4)(X)(2)](BF4)(2) (X = Cl, H) | Not relevant |
| 33 | Chemistry, [Mn(pd)2(gox)] | Not relevant |
| 34 | Chemistry, [Mn(pd)2(H2O)2]+ (Hpd = pentane-2,4-dione) | Not relevant |
| 35 | Chemistry, [Pd-2(L)(4)] | Not relevant |
| 36 | Chemistry, [Pd-2(dppm)(2)(mu-I)(Me)(2)]I | Not relevant |
| 37 | Chemistry, crystalline trans-[Pd(H2O)(2)(NO3)(2)] | Not relevant |
| 38 | Chemistry, [Pd2I2{Te((2.6-OCH3)(2)C6H3)}(2)] (PCy3)(2)] | Not relevant |
| 39 | Chemistry, Pd+2 species | Not relevant |
| 40 | Chemistry, [Zn-2(H2O)(3){PdCl2(pydc)(2)}](n) (Zn-Pd-2) | Not relevant |

**Table S10.** Summary of Web of Science (All Collections) search conducted on August 5, 2025 using the refined search string. The total number of search results reported includes preprints and all document types in the English language.

| **Search String** | **Number of search results (Web of Science (All Collections) August 5, 2025)** |
| --- | --- |
| TS=  "6PPD" OR "DBDA" OR "DMBPD" OR "DPPDA"  OR "(4-anilino-phenyl)-(1,3-dimethyl-butyl)-amine" OR "1,4-Benzenediamine" OR "1,4-Benzenediamine, N-(1,3-dimethylbutyl)-N'-phenyl-" OR "1,4-Benzenediamine, N-(1,3-dimethylbutyl)-N′-phenyl- (9CI)" OR "1-N-(4-methylpentan-2-yl)-1-N-phenylbenzene-1,4-diamine"  OR "1-N-(4-methylpentan-2-yl)-4-N-phenylbenzene-1,4-diamine" OR "4-(1,3-Dimethylbutyl)amino-diphenylamine" OR "4-(1,3-dimethylbutylamino)diphenylamine" OR "4-(Dimethylbutylamino)diphenylamine" OR "4-[(4-Methyl-2-pentyl)amino]diphenylamine"  OR "4-N-(4-methylpentan-2-yl)-1-N-phenylbenzene-1,4-diamine" OR "n-(1,3-dimethyl butyl)-n'-phenyl-p-phenylenediamine" OR "N-(1,3-Dimethylbutyl)-N/'-phenyl-p-phenylenediamine" OR "N-(1,3-Dimethylbutyl)-N\'-phenyl-p-phenylenediamine" OR "n-(1,3-dimethylbutyl)-n'-phenyl-1,4-benzenediamine" OR "N-(1,3-Dimethylbutyl)-N'-phenyl-1,4-phenylenediamine"  OR "N-(1,3-dimethylbutyl)-N'-phenylbenzene-1,4-diamine" OR "N-(1,3-Dimethylbutyl)-N'-phenyl-p-phenylenediamine" OR "N-(4-Methyl-2-pentyl)-N-phenyl-1,4-benzenediamine" OR "N-(4-Methyl-2-pentyl)-N-phenyl-1,4-diaminobenzene" OR "N-(4-Methyl-2-pentyl)-N'-phenyl-1,4-phenylenediamine" OR "N-(4-Methyl-2-pentyl)-N'-phenyl-p-phenylenediamine" OR "N1-(1,3-Dimethylbutyl)-N4-phenyl-1,4-benzenediamine"  OR "N1-(1,3-Dimethylbutyl)-N4-phenyl-1,4-benzenediamine (ACI)" OR "N1-(4-methylpentan-2-yl)-N4-phenylbenzene-1,4-diamine" OR "N-1,3-dimethylbutyl-N`-phenyl-p-phenylenediamine" OR "N-1,3-Dimethylbutyl-N'-phenyl-p-phenylendiamine" OR "N-1,3-Dimethylbutyl-N'-phenyl-p-phenylenediamine" OR "N-1,3-dimethylbutyl-N'-phenyl-p-phenylene-diamine"  OR "N-Dimethylbutyl-N'-phenyl-p-phenylendiamine" OR "N-Phenyl-N'-(1,3-dimethyl butyl)-para-phenylenediamine" OR "N-PHENYL-N'-(1,3-DIMETHYL BUTYL)-PARA-PHENYLENEDIAMINE" OR "n-phenyl-n'-(1,3-dimethylbutyl)-1,4-phenylenediamine" OR "N-PHENYL-N'-(1,3-DIMETHYLBUTYL)-P-PHENYL DIAMINE" OR "N-Phenyl-N'-(1,3-dimethylbutyl)-p-phenylenediamine"  OR "p-Phenylenediamine, N-(1,3-dimethylbutyl)-N'-phenyl-" OR "p-Phenylenediamine, N-(1,3-dimethylbutyl)-N′-phenyl- (7CI, 8CI)" OR "2921519090" OR "ISCS number 1635" OR "UN number 3077" OR "50809-58-0" OR "76600-84-5" OR "793-24-8"  OR "AKOS015901311" OR "BRN 2215491" OR "CAS-793-24-8" OR "CCRIS 2352" OR "CCRIS 4801" OR "CHEMBL1558796" OR "CS-W012405" OR "D3331"  OR "DTXCID605114" OR "DTXSID9025114" OR "E76147" OR "EC 212-344-0" OR "EINECS 212-344-0" OR "GG-0240" OR "HJD0U67PS1"  OR "HSDB 5755" OR "MFCD00072248" OR "NCGC00091548-01" OR "NCGC00091548-02" OR "NCGC00091548-03" OR "NCGC00258444-01" OR "NS00003932"  OR "Q-201440" OR "Q27279957" OR "SCHEMBL39447" OR "Tox21_200890" OR "UNII-HJD0U67PS1" OR "UOP 562" OR "UOP 588" OR "Accinox ZC" OR "Akrochem antiozonant pd-2"  OR "Antage 6C" OR "Antigene 6C" OR "Antioxidant 4020" OR "Antioxidant 6C" OR "Antioxidant CD 13" OR "Antioxidant cd" OR "Antioxidant PD 2"  OR "Antozite 67" OR "Antozite 67F" OR "Diafen 13" OR "Diafen FDMB" OR "Dusantox 6PPD" OR "Flexzone 7F" OR "Flexzone 7L" OR "Flexzone 7P" OR "Forte 6C" OR "Kumanox 13"  OR "Kumanox 13C" OR "Luvomaxx 6PPD" OR "Nocceler 6C" OR "Nocrac 6C" OR "Nocrane 6C" OR "Nocrane 7 L" OR "Ozonon 6C" OR "Ozonone 6C"  OR "Permanax 120" OR "Permanax 6PPD" OR "Pilflex 13" OR "Rubatan BF" OR "Santoflex 13" OR "Santoflex 13F"  OR "Santoflex 6PPD" OR "Stangard 6PPD" OR "Sunsine 6PPD" OR "UOP 562" OR "UOP 588"  OR "Vulkanox 4020" OR "Vulkanox 4020LG" OR "Wingstay 300" OR PX-13 OR "CD 13" OR "NCI-C56315"  OR "6PPD*" OR "6PPDQ" OR "6PPD-Q" OR "6PPD-q" OR "6PPD-quinone"  OR "154926030" OR "2-((4-Methylpentan-2-yl)amino)-5-(phenylamino)cyclohexa-2,5-diene-1,4-dione" OR "2,5-Cyclohexadiene-1,4-dione, 2-[(1,3-dimethylbutyl)amino]-5-(phenylamino)-" OR "2-[(1,3-Dimethylbutyl)amino]-5-(phenylamino)-2,5-cyclohexadiene-1,4-dione"  OR "2754428-18-5" OR "DTXSID301034849" OR "G8MFB8G7B6" OR "N-(1,3-Dimethylbutyl)-N′-phenyl-p-phenylenediamine quinone" | 2,397 |

**Text S1**

We conducted a preliminary search in Web of Science (All Collections) with the proposed search terms (acronym + identifier + chemical name + trade name + unknown) listed in Table S2 & S3. This search was conducted on July 26, 2025 and including preprint citations, all document types, and refined to English language only, resulted in 1,518,311 results with a 100% return rate of benchmark articles. The number of results was higher than expected and we conducted several additional searches on July 28 and 29, 2025 with different configurations of the search term categories (Table S5). A large proportion of results were identifiers (99%) followed by trade names (0.5%). We then reviewed the list of identifier search terms; one search term, “CI”, contributed the most to the results. When “CI” was removed from the identifiers list the results were reduced from 1,509,926 to 5. On July 29, 2025, we also searched each individual search trade name (Table S6). Only two trade names produced >50 results, "Antioxidant 4020" and "PD 2". We repeated the search on August 5, 2025, to update the results with a distribution of document types which were reported in Table S6.

On August 4, 2025, additional searches for individual search terms were conducted (“CI”, “PD 2”, "Akrochem antiozonant pd-2", and “*p*-Phenylenediamine, *N*-(1,3-dimethylbutyl)-*N*′-phenyl- (7CI, 8CI)”) to assess the relevancy to the target literature about 6PPD/6PPDQ (Table S7-S9). None of the first 40 titles and abstracts for each search were relevant to this effort.

On August 5, 2025 the Web of Science (All Collections) search for “CI” AND “6PPD” resulted in 4 results in the English language. We also conducted a search for “6PPD*” alone and all 4 results for “CI” AND “6PPD*” were captured within the 715 results reported for “6PPD*” alone. The search for “PD 2” AND “6PPD*” did not return any results. We concluded from this effort to remove “CI” and “PD 2” and remove the repeated terms “UOP 562” and “UOP 588”. The final search term list is reported in Table S10. This search string returned 2,397 English language only results on August 5, 2025 with a 100% return rate for benchmark articles.

**Text S2**

**Map 1 and Map 2 Combined Screening Questions – ‘Title and Abstract’ (and ‘Full text’ – when required (i.e., when ‘Title and Abstract’ screening result is ‘maybe’ decision))**

If the title or abstract do not provide sufficient information to confidently assess, the study is to be included for full text screening.

Full text screening uses the same questions as the ‘Title and Abstract’ screening

**Map 1 – Occurrence of 6PPD and 6PPDQ in the environment and biota**

Aim: To identify the exposure of biota in the environment to 6PPD and 6PPDQ.

**Map 2 – Effects of 6PPD and 6PPDQ on biota**

Aim: To identify the effects of 6PPD and 6PPDQ on living organisms in manipulative experiments

Screening Questions:

1. **Is the article an empirical study (i.e., not a review article, meta-analysis or modeling study without environmental data) published in a journal, MSc or PhD thesis, conference proceedings, book chapter or technical report?** Yes/No; If Yes, proceed to Q2; If No, article is screened out. Review articles, meta-analyses, book chapters and modeling studies without environmental data are not eligible and thus excluded; however, relevant review/meta-analysis/book chapters/modeling article reference lists will be examined for articles that may be relevant to the dataset.
2. **Is the article written in English?** Yes/No; If Yes, proceed to Q3, If No, article is screened out.
3. **Population**: **Is the population living organisms as defined in Table 2?** Yes/No; If Yes, proceed to Q4; If No, proceed to Q8.
4. **Exposure: Does the study contain a manipulative experiment as defined in Table 2?** Yes/No; If Yes, proceed to Q5; If No, proceed to Q8. Exposure routes include water, sediment, internal administration, etc.
5. **Exposure:** **Does the experiment test exposure of living organisms to 6PPD and/or 6PPDQ as defined in Table 2?** If Yes, proceed to Q6; If No, proceed to Q8. Chemical mixtures, such as tire leachate, wastewater or biosolids are not relevant and are to be excluded from the dataset.
6. **Comparator**: **Is a control treatment included in the experiment, where living organisms are not experimentally exposed to 6PPD and/or 6PPDQ?** Yes/No; If Yes, proceed to Q7; If No, proceed to Q8.
7. **Outcome: Does the study report an outcome as defined in Table 2?** Yes/No; If Yes, study is screened into “eligible for Map 2” and proceeds to Q8; If No, proceed to Q8. Effects on living organisms or proxy of living organisms, such as cell lines, at all levels of biological organization are relevant (e.g., molecular to community level).
8. **Population and Outcome: Does the study report 6PPD or 6PPDQ in the environment as defined in Table 1?** If Yes to Q7 and Yes to Q8, study is screened in for “Eligible for both Map 1 and Map 2”. If Yes in Q7 and No in Q8, study is screened in for “Eligible for Map 2”. If No to Q8, study is screened out.
